# Physiological and Transcriptomic Responses of the Freshwater Hydrozoan *Craspedacusta sowerbii* to Acute Antibiotic and Cadmium Exposure

**DOI:** 10.3390/biology15020193

**Published:** 2026-01-21

**Authors:** Hailong Yan, Yu Wang, Yufan He, Jinglong Wang, Mengyao Wu, Jianing Shi, Jingjing Guo, Shang Shi, Nicola Fohrer, Jianguang Qin, Yuying Li

**Affiliations:** 1 International Joint Laboratory of Watershed Ecological Security for Water Source Region of Middle Route Project of South-North Water Diversion in Henan Province, College of Water Resource and Modern Agriculture, Nanyang Normal University, Nanyang 473061, China; 15637509979@163.com (Y.H.); jinglong_w@163.com (J.W.); 2023086001022@nynu.edu.cn (M.W.); 18630773558@139.com (J.S.); 18437928059@163.com (J.G.); ivy_office2020@163.com (S.S.); nfohrer@hydrology.uni-kiel.de (N.F.); 2 Henan Academy of Fishery Sciences, Henan Academy of Agricultural Sciences, Zhengzhou 450044, China; wangyu@hnagri.org.cn; 3 Department of Hydrology and Water Resources Management, Kiel University, 240980 Kiel, Germany; 4 College of Science and Engineering, Flinders University, Adelaide, SA 5001, Australia; jian.qin@flinders.edu.au

**Keywords:** *Craspedacusta sowerbii*, transcriptomic response, water pollution, antibiotic, heavy metal

## Abstract

Chemical contaminants are increasingly detected in freshwater environments, yet the physiological and molecular responses of many non-model freshwater invertebrates to acute chemical stress remain poorly understood. In this study, we investigated the physiological and transcriptomic responses of the freshwater hydrozoan *Craspedacusta sowerbii* to two widespread aquatic pollutants: the antibiotic sulfamethoxazole (20 μM) and the heavy metal salt CdSO_4_ (10 μM). Morphological and behavioral observations showed that sulfamethoxazole exposure led to reduced motility and body shrinkage, whereas cadmium exposure caused rapid loss of movement and complete mortality within 24 h. RNA sequencing revealed distinct transcriptional response patterns to the two stressors. Sulfamethoxazole exposure primarily induced the up-regulation of genes associated with oxidative stress, apoptosis, immune responses, and signaling pathways, suggesting an active but limited stress-adaptation response. In contrast, cadmium exposure resulted in extensive down-regulation of genes involved in metabolic pathways, cell cycle regulation, fatty acid metabolism, and anti-aging processes, suggesting severe disruption of core metabolic processes. Comparative pathway analyses identified both shared stress-related responses and pollutant-specific transcriptional signatures, with cadmium exerting markedly stronger inhibitory effects at both physiological and molecular levels. These results reveal clear thresholds of stress tolerance and response failure in *C. sowerbii* under chemical pollution, and highlight its ecological sensitivity to water quality deterioration. Together, these findings provide mechanistic insight into acute pollutant-induced stress responses in a freshwater Cnidarian and offer a useful reference for understanding how freshwater invertebrates respond to short-term chemical disturbances.

## Simple Summary

Freshwater ecosystems are increasingly threatened by chemical pollution derived from human activities, yet the responses of small and often overlooked invertebrates remain poorly understood. *Craspedacusta sowerbii*, a globally invasive freshwater jellyfish, is widely distributed in human-impacted waters and may be severely affected by environmental stressors. In this study, we examined how *C. sowerbii* responds to two common aquatic pollutants: the antibiotic sulfamethoxazole and the heavy metal cadmium. We found that sulfamethoxazole primarily reduced swimming activity and induced body shrinkage, whereas cadmium caused rapid loss of movement, severe tissue disintegration, and mortality within 24 h. Gene expression analyses revealed that the jellyfish activated stress- and repair-related pathways under antibiotic exposure, while cadmium exposure broadly suppressed metabolic and cell cycle processes, overwhelming physiological defenses. These results demonstrate that different pollutants impose distinct limits on stress tolerance in *C. sowerbii* and highlight its potential value as an early-warning organism for freshwater pollution. Understanding how such organisms respond to chemical stressors is essential for improving our ability to assess ecological risks and interpret organismal responses to freshwater pollution under increasing anthropogenic pressure.

## 1. Introduction

Global environmental challenges are intensifying at an alarming rate, with issues such as climate change, air pollution, land degradation, and water pollution intricately intertwined [1,2]. Pollution problems, arising from a confluence of natural and anthropogenic factors, have inflicted unprecedented damage on the current ecological environment, severely affecting the organisms that inhabit it [3,4]. Among these multifaceted environmental concerns, water pollution stands out as particularly critical, serving as a significant bottleneck to sustainable development [5]. The indiscriminate release of vast amounts of industrial wastewater, agricultural runoff laden with pollutants, and untreated domestic sewage directly into water bodies has precipitated a notable decrease in water quality [6]. Additionally, the substantial influx of pollutants from sewage into aquatic environments not only threatens the safety of drinking water supplies, heightening the risk of disease transmission, but also results in the destruction of vital habitats for aquatic organisms [6,7]. Aquatic biological poisoning incidents are more frequent, further destabilizing fragile aquatic ecosystems. Persistent anthropogenic pressures can disrupt biodiversity and reduce ecosystem stability [7,8,9]. The degraded ecosystem subsequently undermines vital services such as water purification and flood mitigation, perpetuating environmental decline [6,9].

The presence of emerging contaminants and heavy metals within aquatic ecosystems has escalated into a pressing global ecological crisis [10,11]. Alarmingly, millions of tons of these substances have been released into water bodies without adequate treatment [12,13]. Both types of pollutants are pervasive in contaminated aquatic systems, and their detrimental effects propagate through ecosystems via food chain transmission, resulting in far-reaching ecological repercussions [14,15]. Antibiotic pollutants, whether naturally occurring or synthetic, are prime examples of emerging contaminants, infiltrating water bodies through medical wastewater, livestock farming practices, and aquaculture discharges [16,17]. A previous study has shown that tetracycline, an antibiotic produced by Actinomycetes, significantly reduces microbial community diversity in aquatic ecosystems [18]. Moreover, antibiotic pollutants exert toxic effects on the liver, kidneys, and reproductive organs of aquatic organisms [19,20]. In severe cases, they can even lead to mortality, thereby threatening aquatic biodiversity and disrupting the delicate balance of the aquatic environment [20]. In contrast, heavy metal pollution induces more persistent ecological disturbances, leading to malformations and extinctions of organisms, and thus exerts a profound impact on the ecosystem [21]. Elements such as mercury, cadmium, and lead enter water bodies through mining operations, electroplating processes, and industrial wastewater discharges. Subsequently, they accumulate in sediments and become increasingly concentrated as they ascend the food chain [21,22]. The toxicity of heavy metals to aquatic organisms manifests as damage to multiple physiological systems; for instance, cadmium toxicity causes severe damage to the liver and bones and can reduce calcium ion uptake in the body [22]. The accumulation of cadmium can induce tissue-specific variations and severely compromise the integrity of tissue structure and function, as well as the antioxidant defense system and the immune system, in both fish and freshwater cladocerans [23,24]. While extensive research has explored antibiotics and heavy metals, significant knowledge gaps remain regarding the effective mitigation of their ecological effects, particularly the adaptive responses of non-model organisms to these pollutants, which are still poorly understood [25].

*Craspedacusta sowerbii* (Lankester, 1880), a freshwater hydrozoan belonging to the phylum Cnidaria [26,27,28], is originally from the Yangtze River in China, but has since dispersed globally, colonizing every continent except Antarctica, thereby establishing itself as one of the most successful aquatic colonizers [29,30,31]. Despite its widespread distribution, *C. sowerbii* has rarely been the subject of recent research, primarily due to the challenges associated with cultivating it in laboratory settings, sourcing sufficient material from wild populations, and the difficulty of reliably recognizing and identifying the species in natural environments [32]. The successful colonization of *C. sowerbii* largely depends on suitable environmental conditions and the availability of biological resources [33,34]. Although a variety of factors, such as temperature, water quality, food availability, light, and osmotic pressure, can exert an influence on the life cycle stages of *C. sowerbii* either independently or in combination [33,35,36], this organism still demonstrates a remarkable capacity to thrive across a broad spectrum of freshwater conditions, encompassing eutrophic and human-impacted environments [26,32,33]. In some regional contexts, the species has attracted attention due to its episodic medusa blooms and conspicuous appearance; however, at a global scale, it is widely recognized as an invasive and adaptable freshwater hydrozoan, highlighting the importance of understanding its ecological responses to environmental stressors in increasingly human-modified water systems [26,37]. Research efforts have been directed towards elucidating the response mechanisms of *C. sowerbii* when confronted with stressful environments. Notably, a study has revealed that intense light significantly influences the vertical migration patterns of *C. sowerbii*, thereby shaping its position in the water column [36]. Additionally, food scarcity and drought conditions stimulate the formation of a chitin-covered resting stage in *C. sowerbii*, enabling it to endure extreme conditions [27,38]. The survival duration of *C. sowerbii* declines sharply when osmotic pressure exceeds 34 mOsm/L [35]. Through phenotypic plasticity, *C. sowerbii* can adjust its population dynamics in response to varying dietary qualities [39]. Despite the increasing number of reported sightings, our understanding of the genetics, physiology, and ecology of *C. sowerbii* still remains notably limited, highlighted by a significant lack of data regarding its physiological responses to environmental challenges [40,41,42,43].

In this study, *C. sowerbii* was experimentally exposed to two prevalent water pollutants: the antibiotic sulfamethoxazole (SMZ) and the heavy metal salt cadmium sulfate (CdSO_4_, Cd). Subsequently, the physiological responses of *C. sowerbii* were observed. Specimens exhibiting distinct physiological symptoms were collected for transcriptome sequencing to analyze the transcriptional responses of *C. sowerbii* to these water pollutants. The results revealed that both SMZ (20 μM) and Cd (10 μM) significantly impacted the motility of *C. sowerbii*. Notably, exposure to Cd for 6 h resulted in a complete loss of motility, followed by physical disintegration of the organism after 24 h. The transcriptome data provided profound insights into the molecular mechanisms underlying the transcriptional responses of *C. sowerbii* to these two types of water pollutants. A substantial number of genes associated with oxidative stress, pathological signaling pathways, apoptosis, and programmed cell death were up-regulated. Conversely, genes involved in critical biological processes such as the cell cycle, immunity, and anti-aging processes were down-regulated. The primary objective of this study is to characterize the physiological and transcriptomic responses of the freshwater hydrozoan *C. sowerbii* to acute, high-intensity exposure scenarios of chemical pollution, with a particular focus on stress tolerance and the thresholds for response failure. Furthermore, the study provides a mechanistic framework for interpreting how a globally invasive freshwater Cnidarian responds to chemical stressors at both physiological and transcriptomic levels, thereby enhancing our broader understanding of pollutant-induced stress responses in freshwater invertebrates under anthropogenic pressure.

## 2. Materials and Methods

### 2.1. Pharmaceutical Management and Biosafety

SMZ was procured from Sigma-Aldrich (PubChem CID: 329824585). To prepare the stock solution, SMZ powder was accurately weighed using an analytical balance and subsequently added to a 0.4% sodium hydroxide solution. The mixture was stirred thoroughly until the powder was completely dissolved, yielding a stock solution with a concentration of 30 mg/mL. The solution was then filtered through a 0.22 μm filter membrane for sterilization purposes and transferred to a sterile container for future use. CdSO_4_ was obtained from a domestic reagent supplier. The CdSO_4_ powder was precisely weighed and dissolved in deionized water to prepare a stock solution with a concentration of 10 mg/mL. This solution was also filtered through a 0.22 μm filter membrane and transferred to a container for subsequent use. Following the experiments, the culture solutions containing these chemicals were collected and transported to a qualified facility responsible for the proper treatment and disposal of contaminated water.

### 2.2. Cultivation of C. sowerbii and Morphological Observation

Over 450 specimens of *C. sowerbii* were collected from a natural pond [26] and carefully transferred into a 10 L plastic bucket. These specimens were cultured using water drawn directly from the same pond. Subsequently, they were transported to an indoor laboratory for further experimentation. Upon arrival, the specimens were randomly divided into nine groups, with three serving as control groups (CK). The remaining three groups were exposed to the addition of SMZ, at a final concentration of 20 μM (The final culture pH was measured at 7.19), while the last three groups were treated with a final concentration of 10 μM CdSO_4_ (termed Cd). Each group, consisting of approximately 50 individuals, was placed in a 3 L glass aquarium measuring 25 cm in length, 10 cm in width, and 12 cm in height (File S1, Supplementary Figure S1). All the aquaria were then placed inside an incubator, set to maintain a constant temperature of 25 °C. Morphological observations of *C. sowerbii* individuals were conducted using a Canon 80D digital camera equipped with a 50 mm macro lens (Canon Ltd., Tokyo, Japan). The photographs were edited to enhance contrast utilizing software such as Adobe Photoshop (Adobe Systems Incorporated, San Jose, CA, USA). As previously reported, tentacle kinematics and the frequency of swimming movements per minute serve as key indicators for assessing the relative activity dynamics of *C. sowerbii* [44]. Swimming frequency was determined by randomly selecting an individual of *C. sowerbii*, counting its total swimming movements (actively pulsating) over a defined observation period, recording the total duration, and then dividing the total number of movements by the recorded time. The quantitative statistical data in this study are presented as mean ± S.D. (*n* ≥ 10). Student’s *t*-test was employed to conduct significance analysis between groups at identical time points, with groups exhibiting a *p*-value of less than 0.01 being identified as demonstrating statistically significant differences.

### 2.3. Sample Preparation for RNA-Seq Analysis

The experiment comprised nine groups: three CK groups, three SMZ-treated groups, and three Cd-treated groups; each group initially contained approximately 50 individuals of *C. sowerbii*. Following the addition of SMZ or Cd, 15 individuals were randomly sampled from each group at 2, 6, and 24 h post-treatment for subsequent RNA-seq analysis. This sampling strategy generated three biological replicates for each of the nine treatment-time combinations: CK_2 h, CK_6 h, CK_24 h, SMZ_2 h, SMZ_6 h, SMZ_24 h, Cd_2 h, Cd_6 h, and Cd_24 h. Among the collected samples, CK_6 h and CK_24 h showed no significant physiological differences with CK_2 h, SMZ_6 h showed no significant physiological differences with SMZ_2 h, and the individuals of *C. sowerbii* in Cd_24 h showed severe body disintegration. Therefore, a subset comprising CK_2 h, SMZ_2 h, SMZ_24 h, Cd_2 h, and Cd_6 h was selected for RNA-seq analysis to assess transcriptional responses to SMZ and Cd exposures (File S1, Supplementary Figure S1). For each replicate, *C. sowerbii* individuals were carefully picked using a pipette with a diameter of approximately 10 mm, and all 15 *C. sowerbii* individuals were subsequently placed into a 15 mL freezing tube. A rolled-up filter paper was inserted into the freezing tube to absorb any remaining water. The samples were promptly frozen in liquid nitrogen and stored at −80 °C until RNA extraction was performed. The total RNA of these samples was extracted using Trizol reagent (Invitrogen, Carlsbad, CA, USA), strictly adhering to the manufacturer’s instructions. The RNA quantity was then measured using a Nanodrop 2000 spectrophotometer (Thermo Fisher Scientific, Waltham, MA, USA), and RNA integrity was assessed using an Agilent 2100 bioanalyzer system along with an RNA Nano 6000 Assay kit (Agilent Technologies, Santa Clara, CA, USA). Only qualified RNA was reverse-transcribed into double-stranded cDNA, which was subsequently subjected to sequencing, performed in 2 × 150 bp paired-end runs utilizing an Illumina NovaSeq X Plus system (Illumina, San Diego, CA, USA). This entire sequencing process was performed at Novogene Co., Ltd. (Beijing, China).

### 2.4. Bioinformatics Analysis Pipeline

The raw data, presented in fastq format, initially underwent processing using the fastp software (v0.23.4). During this preprocessing stage, clean data were generated by eliminating reads containing adapters, reads with poly-N sequences, and low-quality reads from the raw dataset. All subsequent analyses were conducted based on these high-quality, clean data. Transcriptome assembly of the RNA-seq data was carried out using Trinity (v2.15.1) software. Building upon the transcriptome assembly results produced by Trinity, Corset (v1.09) was utilized to segregate transcripts exhibiting differential expression across samples from their original clusters and to form new clusters. Ultimately, each cluster was designated as a gene. The assembly quality of the Trinity.fasta, unigene.fa, and cluster.fasta files derived from the assembly process was evaluated using the BUSCO software (v3.0.2, Benchmarking Universal Single-Copy Orthologs. File S1, Supplementary Figure S2). Gene functional annotation was performed based on databases including Nr (the protein sequence database of NCBI); Nt (the nucleic acid sequence database of NCBI); Pfam (the most comprehensive classification system for protein domain annotations); KOG (euKaryotic Ortholog Groups); Swiss-Prot (a database curating and studying protein sequences by experienced biologists); GO (an internationally standardized classification system for describing gene functions); and KEGG (a database analyzing the metabolic pathways of gene products and compounds within cells, along with their functions). The transcriptome assembled by Trinity served as the reference sequence (Ref). For each sample, the clean reads were aligned to the Ref. During this alignment process, reads with a mapping quality score below 10 that were successfully mapped as unpaired reads and reads mapped to multiple genomic regions were filtered out. The alignment was performed using the RSEM (v1.3.3) software, incorporating the bowtie2 parameter “mismatch 0” (the default parameter of bowtie2) within RSEM.

### 2.5. Differential Expression Analysis

Differential expression analysis between two groups was conducted using the DESeq2 R package (v1.42.0). DESeq2 provides statistical tools for identifying differential expression in digital gene expression data, employing models based on the negative binomial distribution. The *p*-values obtained were adjusted using the Benjamini-Hochberg method to control the false discovery rate. The criteria for significant differential expression were established as follows: an adjusted *p*-value (padj) ≤ 0.05 and an absolute log_2_ fold-change (log2FC) ≥ 1. Gene Ontology (GO) enrichment analysis of differentially expressed genes (DEG) was performed using the GOseq R package, which corrects for gene length bias. GO terms with an adjusted *p*-value less than 0.05 were considered significantly enriched among DEG. The Kyoto Encyclopedia of Genes and Genomes (KEGG) is a database resource that facilitates the understanding of the high-level functions and utilities of biological systems, such as cells, organisms, and ecosystems, based on molecular-level information, particularly large-scale molecular datasets generated by genome sequencing and other high-throughput experimental technologies (http://www.genome.jp/kegg/, accessed on 24 Sep. 2025). We utilized the KOBAS software (v3.0) to statistically assess the enrichment of DEG in KEGG pathways. GO/KEGG pathway names are used here as annotation labels derived from cross-species databases. They do not imply the presence of vertebrate-specific physiological systems in *C. sowerbii*, but rather reflect conserved molecular modules involved in stress response.

## 3. Results

### 3.1. The Symptoms and Features of C. sowerbii Under Water Pollution

**Figure 1:**
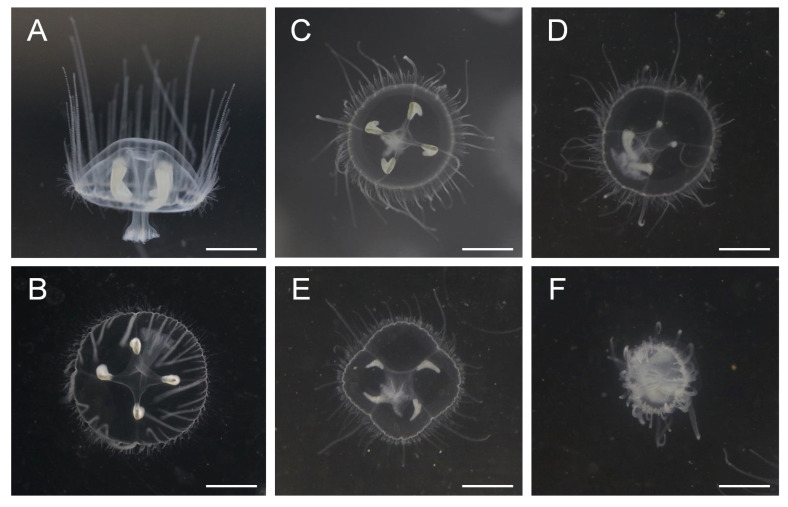
Visual representation of typical *C. sowerbii* during the cultivation period under various treatment conditions. Anterior (**A**) and apical view (**B**) of the specimens in the CK groups. Apical view of the SMZ_2 h groups (**C**), SMZ_24 h groups (**D**), Cd_2 h groups (**E**), and Cd_6 h groups (**F**). It should be noted that *C. sowerbii* individuals remain suspended in the water in (**A**–**C**), whereas they sink to the bottom of the water and exhibit body shrinkage in (**D**–**F**). Bars = 0.5 cm.

In this study, specimens of *C. sowerbii* were cultivated indoors under various pollutant treatments for 24 h, and their relative activity dynamics were recorded at 1, 2, 6, 12, and 24 h post-treatment (File S1, Supplementary Figure S1). In the CK groups, the individuals exhibited continuous vertical swimming, remaining suspended in the water column. The frequency of swimming movements remained stable throughout the observation period, with no discernible differences in individual morphology (Table 1). The tentacle activity of *C. sowerbii* in this treatment was relatively high, with tentacles remaining spread out and positioned above the body, regardless of whether observed from the anterior or apical view (Figure 1A,B). In the SMZ-treated groups, the initial frequency of swimming movements remained relatively stable (Table 1), with no obvious differences in individual morphology observed within the first 12 h (Figure 1C). However, over time, about 24 h post-SMZ treatment, the swimming movement frequency in this group declined to approximately 54% of that in the CK groups (Table 1, *p* < 0.01). After 24 h of cultivation, notable abnormal behaviors (*p* < 0.01) and delayed morphological responses were evident in individual specimens of the SMZ-treated groups. Some individuals began to sink to the bottom of the water and exhibited signs of body shrinkage (Figure 1D). In the Cd-treated groups, no obvious differences in individual morphology were observed within the first 1 h post-treatment (Table 1). However, the swimming movement frequency of *C. sowerbii* began to decline after just 2 h of Cd treatment (Table 1, *p* < 0.01), and the movement ceased entirely by the 12 h mark (Table 1). The body of *C. sowerbii* started to shrink within just 2 h, and this shrinkage became increasingly pronounced as time progressed (Table 1). Eventually, by the 6 h mark, nearly every *C. sowerbii* individual in the Cd-treated groups had curled into a compact mass, with all movement ceasing (Figure 1F). Subsequently, the bodies disintegrated into fragments after 24 h of Cd treatment (File S1, Supplementary Figure S3).

### 3.2. Transcriptomic Variations of C. sowerbii Under Water Pollution

**Figure 2:**
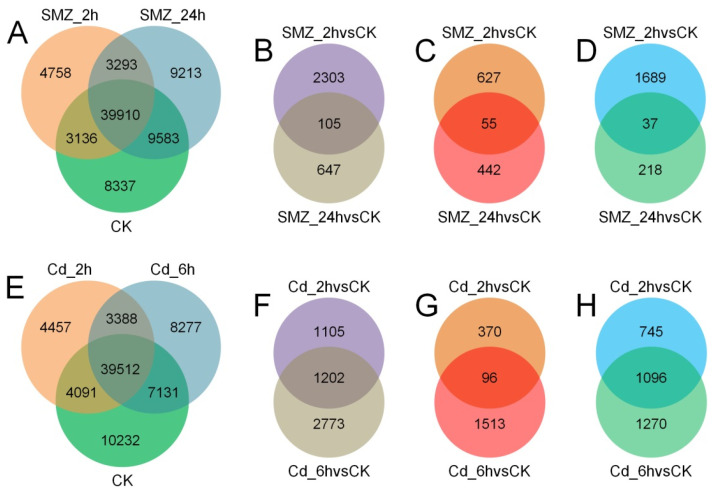
Overview of the transcriptomic variations of *C. sowerbii* in response to water pollution. SMZ-treated groups with co-expression (**A**), differential expression (**B**), up-regulation (**C**), and down-regulation (**D**) patterns. Cd-treated groups with co-expression (**E**), differential expression (**F**), up-regulation (**G**), and down-regulation (**H**) patterns.

The SMZ_2 h and Cd_2 h groups represent the initial stages when *C. sowerbii* begins to respond to water pollutants, and the SMZ_24 h and Cd_6 h groups mark the later stages of its response to these pollutants. Specimens from these groups were collected to analyze the transcriptomic variations of *C. sowerbii* under different types of water pollution, with the CK_2 h (termed CK in RNA-seq analysis) groups serving as the control. A total of 60,966, 51,097, 61,999, 51,448, and 58,308 genes were identified through RNA-seq analysis in the CK, SMZ_2 h, SMZ_24 h, Cd_2 h, and Cd_6 h groups, respectively (Figure 2A,E).

Under SMZ pollution conditions, a total of 43,046, 49,493, and 43,203 genes were co-expressed in the comparisons of SMZ_2 h vs. CK, SMZ_24 h vs. CK, and SMZ_24 h vs. SMZ_2 h groups, respectively (Figure 2A). Among these, 2408 and 752 genes exhibited differential expression in the SMZ_2 h vs. CK and SMZ_24 h vs. CK groups, respectively (Figure 2B, File S2). Specifically, 682 and 497 genes were up-regulated (Figure 2C), while 1726 and 255 genes were down-regulated (Figure 2D) in these comparisons. Notably, a total of 39,910 genes were co-expressed across the CK, SMZ_2 h, and SMZ_24 h groups (Figure 2A). Of these, 105 genes were differentially expressed (Figure 2B), including 55 up-regulated (Figure 2C) and 37 down-regulated genes (Figure 2D).

Upon exposure to Cd pollution, co-expression analysis indicated that 43,603, 46,643, and 42,900 genes were commonly expressed in the pairwise comparisons of Cd_2 h vs. CK, Cd_6 h vs. CK, and Cd_6 h vs. Cd_2 h groups, respectively (Figure 2E). Among the analyzed genes, 2307 and 3975 genes exhibited differential expression patterns in the Cd_2 h vs. CK and Cd_6 h vs. CK groups, respectively (Figure 2F, File S2). Specifically, 466 and 1609 genes were up-regulated (Figure 2G), while 1841 and 2366 genes were down-regulated (Figure 2H) in these comparisons. Notably, a core set of 39,512 genes was consistently co-expressed across the CK, Cd_2 h, and Cd_6 h groups (Figure 2E). Of these, 1202 genes displayed differential expression (Figure 2F), with 96 up-regulated (Figure 2G) and 1096 down-regulated genes (Figure 2H).

### 3.3. Metabolic Pathway Alterations in C. sowerbii in Response to Water Pollution

The DEG are likely of critical importance, playing roles in the metabolic processes that enable *C. sowerbii* to respond effectively to water pollution. Consequently, GO and KEGG analyses were performed on these genes (File S2), aiming to thoroughly investigate the potential functions associated with the top 20 most notably altered terms. However, significant alterations in GO and KEGG terms were observed only in the comparison groups of SMZ_2 h vs. CK and Cd_6 h vs. CK (File S3).

**Figure 3:**
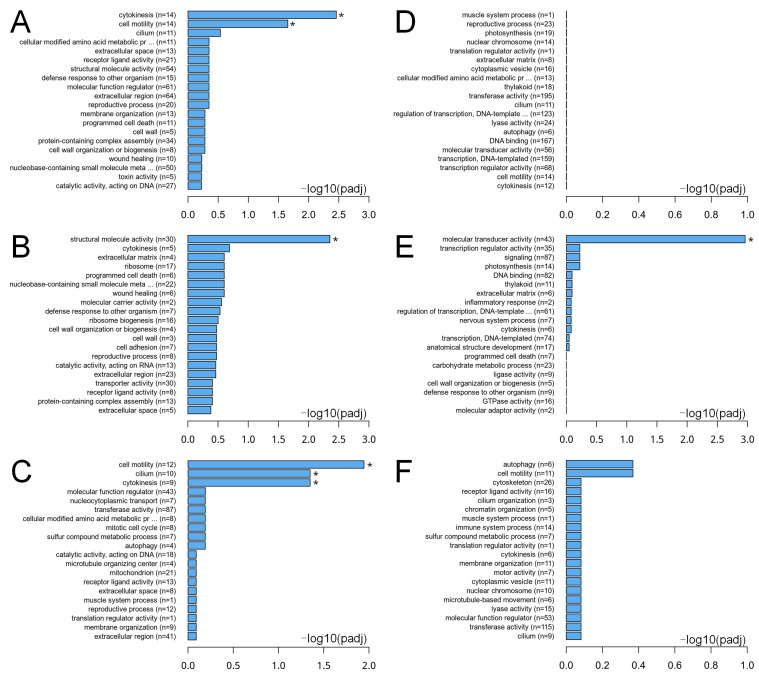
GO analysis of DEG in *C. sowerbii* across different comparison groups. The top 20 GO terms for the overall (**A**), up- (**B**), and down- (**C**) regulated functional pathways in the SMZ_2 h vs. CK groups. The top 20 GO terms for the overall (**D**), up- (**E**), and down- (**F**) regulated functional pathways in the Cd_6 h vs. CK groups. An adjusted *p*-value < 0.05 was considered significantly (*) enriched among DEG.

Under GO analysis, the pathways of SMZ_2 h groups exhibited notable changes compared to the CK groups. Specifically, pathways related to cytokinesis (involving 14 genes) and cell motility (also involving 14 genes) showed significant overall alterations (Figure 3A). Among these, pathways associated with structural molecule activity (involving 30 genes) demonstrated a marked up-regulation (Figure 3B). Conversely, pathways related to cell motility (12 genes), cilium (10 genes), and cytokinesis (9 genes) displayed significant down-regulation (Figure 3C). In the comparison between the Cd_6h and CK groups, although no functional pathways exhibited significant overall alteration (Figure 3D) or significant down-regulation (Figure 3F), pathways associated with molecular transducer activity (encompassing 43 genes) demonstrated significant up-regulation (Figure 3E). It is particularly noteworthy that, in addition to the markedly altered pathways, pathways related to signaling, defense response, programmed cell death, and wound healing were among the most significantly up-regulated. In contrast, pathways associated with molecular activity, cell cycle progression, cell motility, and immune system processes were among the most notably down-regulated in *C. sowerbii* when exposed to water pollutants (Figure 3). 

**Figure 4:**
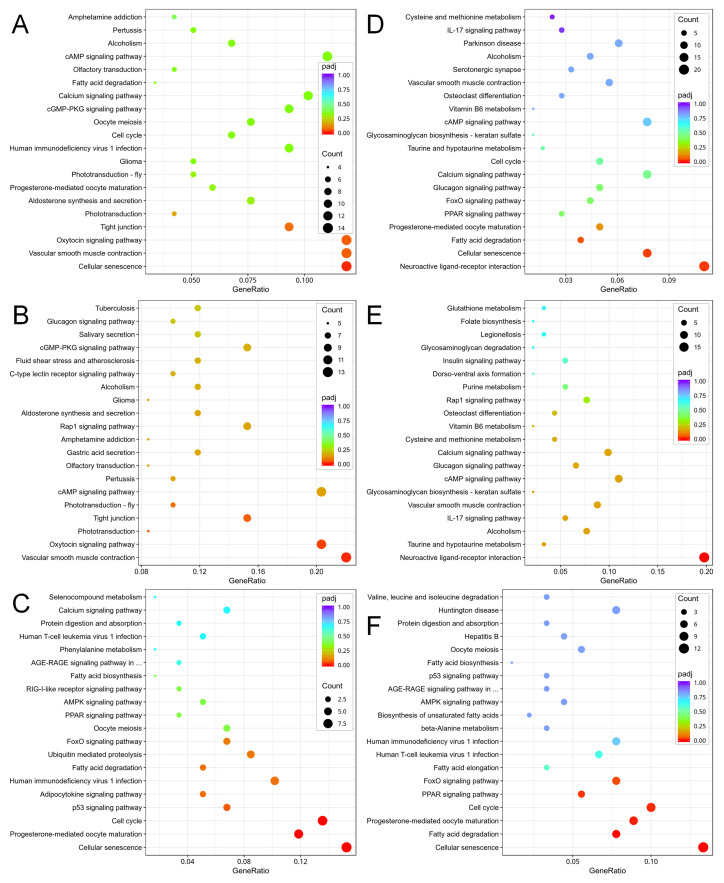
KEGG functional enrichment analysis of DEG in *C. sowerbii* across diverse comparison groups. The top 20 KEGG terms for the overall (**A**), up- (**B**), and down- (**C**) altered functional pathways in the SMZ_2 h vs. CK groups. The top 20 KEGG terms for the overall (**D**), up- (**E**), and down- (**F**) altered functional pathways in the Cd_6 h vs. CK groups.

Under KEGG analysis, distinct patterns of pathway alterations emerged in the comparison groups of *C. sowerbii*. Results indicated that in the SMZ_2 h vs. CK groups, pathways associated with cellular senescence, encompassing 14 genes, demonstrated significant overall changes (Figure 4A). Among these top-regulated pathways, the phototransduction pathway (5 genes), the oxytocin signaling pathway (12 genes), and the vascular smooth muscle contraction pathway (13 genes) exhibited notable up-regulation (Figure 4B). Conversely, pathways related to the cell cycle (9 genes), progesterone-mediated oocyte maturation (7 genes), and cellular senescence (9 genes) showed significant down-regulation (Figure 4C). When comparing the Cd_6 h and CK groups, pathways linked to fatty acid degradation (7 genes), cellular senescence (14 genes), and neuroactive ligand-receptor interaction (20 genes) displayed significant overall alterations (Figure 4D). Notably, the neuroactive ligand–receptor interaction pathway (18 genes) showed marked up-regulation (Figure 4E). In contrast, pathways related to the FoxO signaling pathway (7 genes), the PPAR signaling pathway (5 genes), cell cycle (9 genes), progesterone-mediated oocyte maturation (8 genes), fatty acid degradation (7 genes), and cellular senescence (12 genes) all exhibited significant down-regulation (Figure 4F). Other metabolic pathways that underwent the most significant changes under KEGG analysis included signaling and cell cycle (Figure 4). Although these pathways merely serve as reference metabolic processes in transcriptome analysis, the activation or suppression of these pathways may indicate the cellular and molecular damage inflicted by water pollutants, assisting us in comprehending what reactions will occur in *C. sowerbii* when confronted with pollutants.

### 3.4. Alterations in Gene Expression Profiles of C. sowerbii in Response to Water Pollution

Based on the observed symptoms and the alterations in the functional pathways of *C. sowerbii* under water pollution conditions, particular attention has been directed towards examining the gene expression patterns within key functional pathways under KEGG enrichments (File S2), which encompass cell cycle, cellular senescence, apoptosis, oxidative phosphorylation, FoxO signaling pathway, and the Ras-Rap1 signaling.

**Figure 5:**
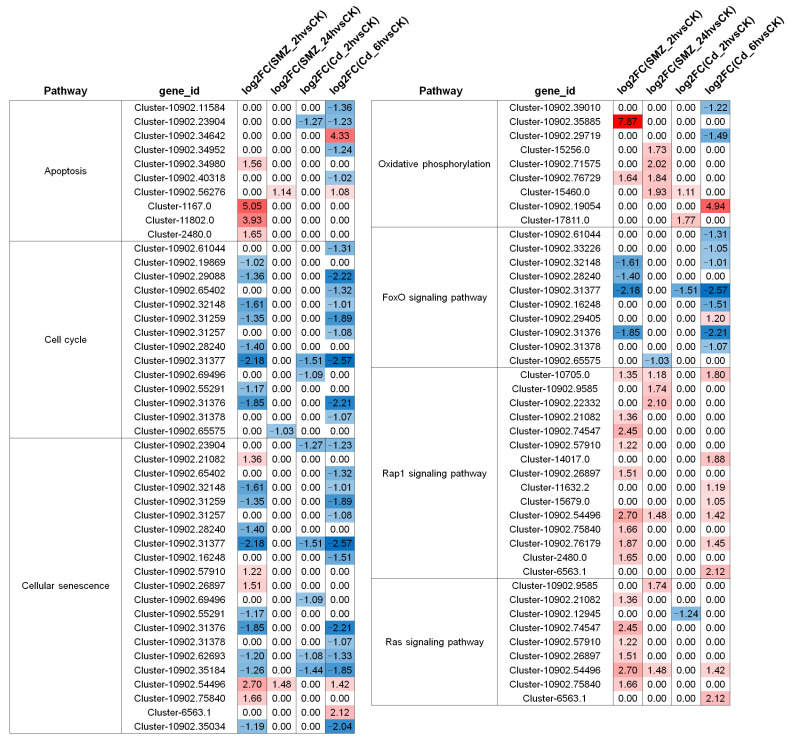
Impact of water pollutants on gene expression patterns in *C. sowerbii*. Differential gene expression was visualized using log2FC values. Genes exhibiting significant up-regulation were highlighted in red, while down-regulated genes were marked in blue across experimental groups. A log2FC value of 0 indicated no differential expression of the gene.

A total of ten apoptosis-related genes were identified, with four demonstrating up-regulation in the SMZ_2 h vs. CK comparisons. In the Cd_6 h vs. CK groups, two genes were up-regulated while four were down-regulated. Notably, the comparisons of SMZ_24 h vs. CK and Cd_2 h vs. CK exhibited only limited expression alterations (Figure 5). Additionally, 14 cell cycle-related genes were identified, all of which consistently demonstrated down-regulation across treatment groups (Figure 5). Among the 21 cellular senescence-associated genes, the majority exhibited down-regulated expression patterns (Figure 5). Furthermore, nine genes involved in oxidative phosphorylation were identified, with most showing up-regulation. Specifically, Cluster-10902.35885 and Cluster-10902.19054 exhibited particularly striking log2FC values of 7.87 and 4.94 in the SMZ_2 h vs. CK and Cd_6 h vs. CK groups, respectively (Figure 5). In the FoxO signaling pathway, ten DEGs were identified, and with the exception of one gene showing a log2FC of 1.20 in the Cd_6 h vs. CK group, all others exhibited down-regulation (Figure 5). Lastly, in the Ras-Rap1 signaling pathway, 24 genes were identified, the majority of which displayed up-regulated expression patterns (Figure 5).

## 4. Discussion

### 4.1. Similarities and Differences of C. sowerbii Under Different Water Pollution Treatments

When confronted with environmental pollutants, certain organisms can gradually acclimate to their surroundings by modulating their physiological metabolism, while others are unable to effectively counteract the physiological toxicity induced by these pollutants, ultimately leading to individual mortality [23,45]. Among the most frequently abused pollutants pervasive in various aquatic environments are the antibiotic SMZ and the heavy metal Cd [10,46]. The concentration of SMZ in aquatic environments can range from 0.3 to 4330 ng/L [47]. The concentration of Cd in freshwater environments can reach approximately 15 μg/L [24]. Though these contaminants occur naturally at low concentrations, human activities significantly increase their levels in the environment [48]. Studies have demonstrated that the extensive use of SMZ has contaminated aquatic ecosystems, resulting in oxidative damage and immune system malfunctions in the livers and gills of organisms; for example, exposure to SMZ at concentrations ranging from 1 to 10 mg/L has been shown to affect the heart rate and swimming behavior of zebrafish [49,50]. The toxicity of Cd to aquatic animals is lethal, and exposure of zebrafish larvae to Cd at concentrations of 3–8 mg/L significantly elevated the mortality rate, accompanied by a notable increase in the expression of heat shock proteins [51,52]. Exposure of other Cnidarians to antibiotics and heavy metals has also been proven to alter their metabolic processes [53,54,55,56]. Under the 5 mg/L tetracycline treatment, the metabolic process of the moon jellyfish *Aurelia aurita* showed functional reorganization triggered by the disruption of the symbiotic microbial community structure [57]. Exposure of the soft coral *Sarcophyton trocheliophorum* to the antibiotic doxycycline hydrochloride at concentrations ranging from 1 to 10 mg/L induced disruption of protein synthesis and ultimately resulted in mortality and bleaching phenomena [58]. RNA-Seq analysis revealed rapid metabolic alterations in the sea anemone *Nematostella vectensis* within just one hour of exposure to heavy metals [59]. Exposure of the jellyfish *Pelagia noctiluca* to heavy metals at concentrations of 2–10 mM resulted in significant inhibition of both nematocyst discharge response and the hemolytic activity of crude venom [60].

Given the scarcity of established research paradigms from previous studies on *C. sowerbii* to serve as references, we primarily drew upon the aforementioned concentration gradients employed in zebrafish experiments or other Cnidarians when carrying out our investigation. In this study, the freshwater hydrozoan *C. sowerbii* was exposed to SMZ and Cd at concentrations of 20 μM (approximately 5 mg/L) and 10 μM (approximately 2.5 mg/L), respectively (The pollutant concentrations employed in this experiment exceeded those typically detected in natural freshwater environments, as this study was designed to examine the acute physiological and transcriptomic effects of accidental discharges or episodic contamination). Morphological observations revealed abnormal behavioral alterations in *C. sowerbii* following treatment with SMZ or Cd for varying durations. These changes primarily manifested as a decrease in swimming activity (Table 1) and symptoms such as body shrinkage (Figure 1). In severe cases, particularly after 24 h of Cd treatment, individuals of *C. sowerbii* succumbed, and their bodies disintegrated (File S1, Supplementary Figure S3), suggesting that these water pollutants were detrimental to the growth and survival of *C. sowerbii*.

The physiological toxicities of these two water pollutants on *C. sowerbii* exhibit both similarities and differences. Phenotypically, both pollutants affect the movement of *C. sowerbii*. However, while individuals treated with SMZ show reduced motility, those subjected to Cd exposure experience not only declines in swimming ability but also the threat of death and disintegration (Figure 1). Furthermore, RNA-seq analysis indicated that when *C. sowerbii* is exposed to SMZ pollution at a later stage, the metabolic response primarily involves the up-regulation of DEG (Figure 2). In contrast, when confronted with Cd pollution, a greater number of DEG exhibit down-regulation patterns (Figure 2). Moreover, the performance of *C. sowerbii* varies at different time points following exposure to different pollutants. Two hours after SMZ treatment, there are 2408 DEG, whereas after 24 h of SMZ exposure, the number of DEG decreases to only 752 (Figure 2). This suggests that *C. sowerbii* rapidly initiates gene transcriptional regulatory responses when exposed to SMZ pollution, thereby conferring a certain degree of resistance to SMZ stress. This observation is also supported by the absence of lethal outcomes in *C. sowerbii* at the phenotypic level (Table 1, Figure 1). However, the scenario markedly differs when *C. sowerbii* is exposed to Cd. Firstly, two hours after Cd exposure, there are 2307 DEGs, a number that increases to 3975 six hours post-exposure (Figure 2). This suggests that, at the transcriptional level, gene regulation in *C. sowerbii* becomes increasingly intense. Secondly, following Cd treatment, the DEG of *C. sowerbii* predominantly exhibit a down-regulation pattern (Figure 2), suggesting that many pathways are inhibited and normal ones are inevitably disrupted. This might explain why *C. sowerbii* ceases movement and begins to disintegrate after Cd treatment.

**Figure 6:**
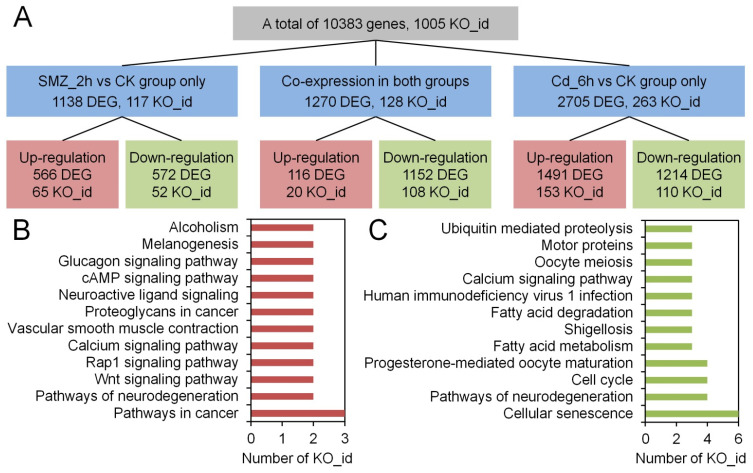
Overview of the transcriptomic variations of *C. sowerbii* in response to water pollutants. The composition of DEG and their corresponding KO_id across various comparison groups (**A**), along with the distribution patterns of KO_id exhibiting up-regulated (**B**) and down-regulated (**C**) expression patterns among the DEG that were commonly identified in both the SMZ_2 h vs. CK groups and the Cd_6 h vs. CK groups.

Transcriptomic data provide a robust approach to characterizing metabolic alterations in organisms in response to pollution stress, thereby elucidating the hazards that pollutants pose to living organisms [61,62]. In this study, the most significant metabolic regulation in *C. sowerbii* was observed in the SMZ_2 h vs. CK and Cd_6 h vs. CK treatment groups (Figure 3, Figure 4 and Figure 5). Consequently, a detailed analysis of the transcriptomic data from these two treatment groups was performed. The results indicated that a total of 10,383 genes were co-expressed in both the SMZ_2 h vs. CK and Cd_6 h vs. CK groups, with 1005 genes possessing a KEGG orthology identity (KO_id) (File S2, Figure 6A). Among all identified genes, 1270 DEG were shared between these two groups, encompassing 128 KO_id (File S2). Of these 1270 DEG, 116 exhibited an up-regulation pattern (with 20 KO_id), while 1152 displayed a down-regulation pattern (with 108 KO_id) (Figure 6A). The up- and down-regulated genes were subsequently enriched based on their KO_id (Figure 6B,C). The results revealed that the top 12 most up-regulated KEGG orthologs were predominantly enriched in pathways linked to cancer, neurodegeneration, Wnt/Rap1/calcium signaling, vascular smooth muscle contraction, proteoglycans in cancer, neuroactive ligand, cAMP, glucagon signaling, melanogenesis, and alcoholism (Figure 6B). These pathways cover a broad range of biological areas and converge on shared mechanisms regulating cell fate [63,64]. In contrast, the top 12 most significantly down-regulated KEGG orthologies were enriched in pathways related to cellular senescence, neurodegeneration, cell cycle, progesterone-mediated oocyte maturation, fatty acid metabolism, shigellosis, fatty acid degradation, human immunodeficiency virus 1 infection, calcium signaling, oocyte meiosis, motor proteins, and ubiquitin-mediated proteolysis (Figure 6C). These pathways predominantly represent interconnected networks that regulate cell cycle progression, stress response, and survival [65,66,67]. It should be noted that functional annotation and pathway enrichment in this study were performed based on cross-species databases, primarily derived from model organisms. KEGG pathway names are used here as annotation labels rather than evidence for the literal presence of vertebrate-specific biological systems in *C. sowerbii*.

**Figure 7:**
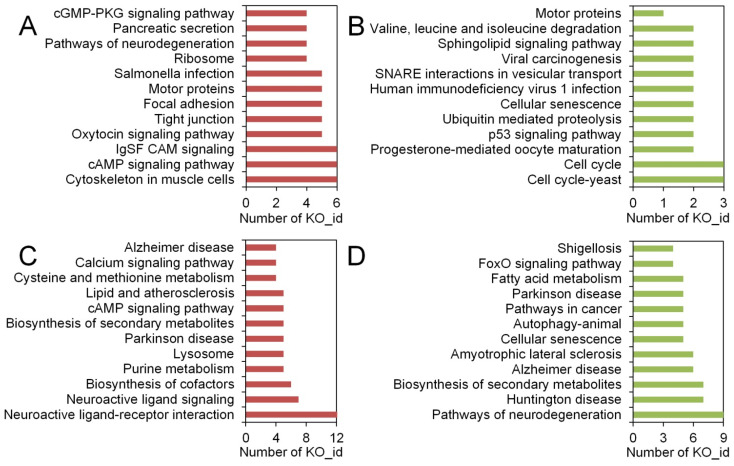
The distribution patterns of KO_id uniquely presented in different groups: Up-regulated (**A**) and down-regulated (**B**) patterns in the SMZ_2 h vs. CK groups only. Up-regulated (**C**) and down-regulated (**D**) patterns in the Cd_6 h vs. CK groups only.

On the other hand, notable differences were observed in the gene expression profiles of *C. sowerbii* under different water pollution treatments. Among the 10,383 identified genes, 1138 DEG were uniquely present in the SMZ_2 h vs. CK groups, encompassing 117 KO_id. Of these 1138 DEG, 566 exhibited an up-regulation pattern (with 65 KO_id), while 572 displayed a down-regulation pattern (with 52 KO_id) (Figure 6A, File S2). Additionally, another 2705 DEG were uniquely identified in the Cd_6 h vs. CK groups, encompassing 263 KO_id. Among these 2705 DEG, 1491 showed an up-regulation pattern (with 153 KO_id), whereas 1214 exhibited a down-regulation pattern (with 110 KO_id) (Figure 6A, File S2). All these up- and down-regulated genes were subsequently enriched based on their KO_id (Figure 7). Notably, pathways in cAMP signaling (up-regulation) and cellular senescence (down-regulation) were enriched not only in the shared gene portions between the SMZ_2 h vs. CK and Cd_6 h vs. CK groups (Figure 6), but also in gene portions unique to either the SMZ_2 h vs. CK groups or the Cd_6 h vs. CK groups (Figure 7). Apart from these two pathways, the remaining pathways exhibited distinct enrichment patterns (Figure 7). This suggests that when subjected to different water pollution treatments, gene expression at the transcriptome level in *C. sowerbii* displays both similarities and differences. These similarities give rise to analogous physiological responses in *C. sowerbii* under the two separate pollution scenarios. Conversely, the differences provide an explanation for why SMZ treatment merely impacts the motility of *C. sowerbii*, while Cd treatment causes their mortality due to its toxic effects.

### 4.2. Adaptation and Evolution of Aquatic Life Under the Stress of Water Pollution

The proliferation of *C. sowerbii* depends on the environmental conditions of aquatic habitats [26,32,33]. However, under the prevalent situation of water pollution, how this species adapts and responds to contamination remains largely unknown. Currently, *C. sowerbii* is globally distributed and displays a tendency to expand its range further due to climate change [29,30,68]. The success of its colonization can be attributed to multiple factors. Research has demonstrated that activities such as fish stocking, bird migrations, and the importation of ornamental aquatic plants and pets facilitate the efficient spread of *C. sowerbii* and significantly increase opportunities for transmission [31,69,70]. Furthermore, *C. sowerbii* employs evolutionary strategies to thrive in adverse conditions, including adopting stress-resistant forms or developing chitinous resting stages to endure harsh environmental circumstances [38,71]. Despite these adaptations, the impact of water pollutants on *C. sowerbii*’s survival remains a critical concern. In this study, it has been discovered that pollutants like SMZ and Cd directly interfere with the behavior and gene expression patterns of *C. sowerbii*, inflicting toxic impacts on individual specimens. Moreover, these contaminants are hypothesized to indirectly hinder the reproductive processes of *C. sowerbii* by modifying the dynamics of aquatic ecosystems. For example, they have the potential to disrupt algal, zooplankton, or microbial communities, consequently resulting in a scarcity of vital nutritional resources that are indispensable for the survival of *C. sowerbii* [19,21,26]. Taking *C. sowerbii* as the model organism and SMZ and Cd as typical pollutants, this study delves into the toxicological effects that common environmental contaminants have on aquatic life. It aims not only to heighten our awareness of the ecological consequences brought about by pollution but also to motivate us to take an active part in environmental protection. This study also provides foundational insights into the physiological and molecular adaptive mechanisms of *C. sowerbii* when exposed to water pollutants, which enhance our understanding of *C. sowerbii*’s evolutionary strategies and offer critical references for other aquatic organisms facing similar pollution challenges.

Despite the advancements made in elucidating the adaptive responses of *C. sowerbii* to water pollution, several limitations remain. First, the research primarily focused on the short-term effects of two isolated pollutants (SMZ and Cd), leaving the long-term consequences of chronic, multi-pollutant exposure unresolved. Future studies should investigate the synergistic interactions among pollutants and their cumulative impacts on the physiological and ecological functions of *C. sowerbii*. Second, while transcriptomic analyses were predominant in this work, the limited integration of proteomic and metabolomic approaches restricts holistic insights into the molecular regulatory networks. Multi-omics strategies, combined with molecular biological techniques such as real-time quantitative PCR and proteinase activity verifications, should be prioritized to unravel the species’ adaptive mechanisms more comprehensively. Third, due to the single sampling site, the transcriptomic data in this study may be subject to errors arising from inconsistent genetic backgrounds and environmental plasticity among *C. sowerbii* individuals. Subsequent research could mitigate this limitation by collecting samples from multiple locations, thereby rendering the research data more objective. Finally, laboratory-based experiments may not fully replicate natural conditions, potentially skewing the results. Strengthening field monitoring alongside controlled studies will enhance ecological relevance, providing a more robust scientific foundation for environmental stewardship and sustainable management.

## 5. Conclusions

This study characterized the physiological and transcriptomic responses of the freshwater hydrozoan *C. sowerbii* to acute exposure to the antibiotic SMZ and the heavy metal Cd. The results revealed marked differences in stress response patterns, with SMZ inducing limited physiological and stress-adaptive transcriptional responses, whereas Cd exposure caused rapid physiological collapse accompanied by broad suppression of metabolic and cell cycle pathways. These findings demonstrate distinct thresholds of stress tolerance and response failure under different classes of chemical pollutants, and provide mechanistic insight into how acute contamination can disrupt organismal function in freshwater Cnidarians.

## Figures and Tables

**Table 1:** The number of swimming movements per minute, which indicates the relative activity dynamics of *C. sowerbii*, during the cultivation time period under various treatments. Quantitative data are presented as mean ± S.D. (*n* ≥ 10). Student’s *t*-test was used for group-to-group significance analysis, and significant differences (**) were identified when *p* < 0.01.

Group	1 h	2 h	6 h	12 h	24 h
CK	42.3 ± 8.1	43.9 ± 5.6	45.5 ± 6.0	43.1 ± 7.2	43.5 ± 6.4
SMZ	43.9 ± 10.8	41.7 ± 13.8	40.4 ± 12.3	41.8 ± 7.5	23.4 ± 8.7 **
Cd	42.7 ± 6.9	20.1 ± 8.3 **	4.3 ± 5.0 **	0	0

## Data Availability

The data are contained within the article.

## Supplementary Materials

The following supporting information can be downloaded at: https://www.mdpi.com/article/10.3390/biology15020193/s1, File S1. Supplementary Figure S1. Schematic diagram illustrating the experimental sampling strategy. Figure S2. The BUSCO assessment result. Figure S3. Following 24 h of Cd treatment, the bodies of *C. sowerbii* ceased movement and disintegrated into fragments. File S2. Raw Data of the DEG. File S3. GO and KEGG functional enrichment.

## References

[1] van Vliet M.T.H., Thorslund J., Strokal M., Hofstra N., Flörke M., Ehalt Macedo H., Nkwasa A., Tang T., Kaushal S.S., Kumar R. (2023). Global river water quality under climate change and hydroclimatic extremes. Nat. Rev. Earth Environ..

[2] Manca F., Benedetti-Cecchi L., Bradshaw C.J.A., Cabeza M., Gustafsson C., Norkko A.M., Roslin T.V., Thomas D.N., White L., Strona G. (2024). Projected loss of brown macroalgae and seagrasses with global environmental change. Nat. Commun..

[3] Kaijser W., Musiol M., Schneider A.R., Prati S., Brauer V.S., Bayer R., Birk S., Brauns M., Dunne L., Enss J. (2025). Meta-analysis-derived estimates of stressor–response associations for riverine organism groups. Nat. Ecol. Evol..

[4] Xie J., Wang T., Zhang P., Zhang H., Wang H., Wang K., Zhang M., Xu J. (2024). Effects of multiple stressors on freshwater food webs: Evidence from a mesocosm experiment. Environ. Pollut..

[5] Jiang X., Kirsten K.L., Qadeer A. (2025). Contaminants in the Water Environment: Significance from the Perspective of the Global Environment and Health. Water.

[6] Yu K., Mohapatra S., Chen Y., Jiang P., Tong X. (2025). Interactive Effects of Climate Change and Contaminants in Aquatic Ecosystems on Environmental-Human Health. Curr. Pollut. Rep..

[7] Rajak P., Ganguly A., Nanda S., Mandi M., Ghanty S., Das K., Biswas G., Sarkar S., Shit P.K., Datta D.K., Bera B., Islam A., Adhikary P.P. (2024). 14-Toxic contaminants and their impacts on aquatic ecology and habitats. Spatial Modeling of Environmental Pollution and Ecological Risk.

[8] Madesh S., Gopi S., Sau A., Rajagopal R., Namasivayam S.K.R., Arockiaraj J. (2024). Chemical contaminants and environmental stressors induced teratogenic effect in aquatic ecosystem—A comprehensive review. Toxicol. Rep..

[9] Jan S., Mishra A.K., Bhat M.A., Bhat M.A., Jan A.T. (2023). Pollutants in aquatic system: A frontier perspective of emerging threat and strategies to solve the crisis for safe drinking water. Environ. Sci. Pollut. Res..

[10] Arambawatta-Lekamge S.H., Pathiratne A., Rathnayake I.V.N. (2021). Sensitivity of freshwater organisms to cadmium and copper at tropical temperature exposures: Derivation of tropical freshwater ecotoxicity thresholds using species sensitivity distribution analysis. Ecotoxicol. Environ. Saf..

[11] Wang F., Xiang L., Sze-Yin Leung K., Elsner M., Zhang Y., Guo Y., Pan B., Sun H., An T., Ying G. (2024). Emerging contaminants: A One Health perspective. Innovation.

[12] Bashir I., Lone F.A., Bhat R.A., Mir S.A., Dar Z.A., Dar S.A., Hakeem K.R., Bhat R.A., Qadri H. (2020). Concerns and Threats of Contamination on Aquatic Ecosystems. Bioremediation and Biotechnology: Sustainable Approaches to Pollution Degradation.

[13] Schar D., Klein E.Y., Laxminarayan R., Gilbert M., Van Boeckel T.P. (2020). Global trends in antimicrobial use in aquaculture. Sci. Rep..

[14] Singh B.K., Paul S., Das I., Singha E.R., Giri A. (2025). Global Warming and Emerging Contaminants: Impacts on Aquatic Organisms and Their Responses. Int. J. Environ. Res..

[15] Grenni P., Ancona V., Barra Caracciolo A. (2018). Ecological effects of antibiotics on natural ecosystems: A review. Microchem. J..

[16] Xiong J.-Q., Govindwar S., Kurade M.B., Paeng K.-J., Roh H.-S., Khan M.A., Jeon B.-H. (2019). Toxicity of sulfamethazine and sulfamethoxazole and their removal by a green microalga, *Scenedesmus obliquus*. Chemosphere.

[17] Zhang H., Quan H., Song S., Sun L., Lu H. (2023). Comprehensive assessment of toxicity and environmental risk associated with sulfamethoxazole biodegradation in sulfur-mediated biological wastewater treatment. Water Res..

[18] Peng P., Yan X., Zhou X., Chen L., Li X., Miao Y., Zhao F. (2024). Enhancing degradation of antibiotic-combined pollutants by a hybrid system containing advanced oxidation and microbial treatment, a review. J. Hazard. Mater..

[19] Studziński W., Gackowska A., Kudlek E., Przybyłek M. (2025). Environmental and toxicological aspects of sulfamethoxazole photodegradation in the presence of oxidizing agents. Environ. Sci. Pollut. Res..

[20] Bojarski B., Kot B., Witeska M. (2020). Antibacterials in Aquatic Environment and Their Toxicity to Fish. Pharmaceuticals.

[21] Galasso F., Frank A.B., Foster W.J. (2025). Heavy metal toxicity and its role as a major driver of past biodiversity crises. Commun. Earth Environ..

[22] Haider F.U., Liqun C., Coulter J.A., Cheema S.A., Wu J., Zhang R., Wenjun M., Farooq M. (2021). Cadmium toxicity in plants: Impacts and remediation strategies. Ecotoxicol. Environ. Saf..

[23] Liu Y., Chen Q., Li Y., Bi L., Jin L., Peng R. (2022). Toxic Effects of Cadmium on Fish. Toxics.

[24] Samarakoon T., Fujino T., Hagimori M., Saito R. (2023). Cadmium uptake and oxidative-stress-induced DNA alterations in the freshwater cladoceran *Moina macrocopa* (Straus 1820) following consecutive short-term exposure assessments. Limnology.

[25] El-Sharkawy M., Alotaibi M.O., Li J., Du D., Mahmoud E. (2025). Heavy Metal Pollution in Coastal Environments: Ecological Implications and Management Strategies: A Review. Sustainability.

[26] Yan H., Wang Y., Wu M., Li Y., Wang W., Zhang D., Guo J., Fohrer N., Li B.L. (2025). Feeding Behavior and Ecological Significance of *Craspedacusta sowerbii* in a Freshwater Reservoir: Insights from Prey Composition and Trophic Interactions. Biology.

[27] Jankowski T. (2001). The freshwater medusae of the world—A taxonomic and systematic literature study with some remarks on other inland water jellyfish. Hydrobiologia.

[28] Acker T.S., Muscat A.M. (1976). The Ecology of *Craspedacusta sowerbii* Lankester, a Freshwater Hydrozoan. Am. Midl. Nat..

[29] Lüskow F., Väinölä R., Lehtiniemi M., von Numers M., Pakhomov E.A. (2025). Evidence for non-indigenous freshwater jellyfish *Craspedacusta sowerbii* spreading in Finland. Hydrobiologia.

[30] Gießler S., Strauss T., Schachtl K., Jankowski T., Klotz R., Stibor H. (2023). Trophic Positions of Polyp and Medusa Stages of the Freshwater Jellyfish *Craspedacusta sowerbii* Based on Stable Isotope Analysis. Biology.

[31] Marchessaux G., Lüskow F., Sarà G., Pakhomov E.A. (2021). Predicting the current and future global distribution of the invasive freshwater hydrozoan *Craspedacusta sowerbii*. Sci. Rep..

[32] Marchessaux G., Bejean M. (2020). From frustules to medusae: A new culture system for the study of the invasive hydrozoan *Craspedacusta sowerbii* in the laboratory. Invertebr. Biol..

[33] Folino-Rorem N.C., Reid M., Peard T. (2016). Culturing the freshwater hydromedusa, *Craspedacusta sowerbii* under controlled laboratory conditions. Invertebr. Reprod. Dev..

[34] Winata K., Zhu J.A., Hanselman K.M., Zerbe E., Langguth J., Folino-Rorem N., Cartwright P. (2024). Life Cycle Transitions in the Freshwater Jellyfish *Craspedacusta sowerbii*. Biology.

[35] Zhang Y.W., Pan X.F., Wang X.A., Jiang W.S., Liu Q., Yang J.X. (2016). Effects of osmotic pressure, temperature and stocking density on survival and sexual reproduction of *Craspedacusta sowerbii*. Dongwuxue Yanjiu.

[36] Lüskow F., Polgári B., Stibor H., Schachtl K., Abonyi A. (2025). Light increases surface occurrence of the freshwater jellyfish *Craspedacusta sowerbii* via positive phototaxis. Hydrobiologia.

[37] Luk C.Y.L. (2024). The Chinese Freshwater Jellyfish Unbound: Evolution, Nomenclature, and Bioinvasion of *Craspedacusta sowerbii*, 1880–1941. Hist. Stud. Nat. Sci..

[38] Gasith A., Gafny S., Hershkovitz Y., Goldstein H., Galil B. (2011). The invasive freshwater medusa *Craspedacusta sowerbii* Lankester, 1880 (Hydrozoa: Olindiidae) in Israel. Aquat. Invasions.

[39] Caputo L., Fuentes R., Woelfl S., Castañeda L.E., Cárdenas L. (2021). Phenotypic plasticity of clonal populations of the freshwater jellyfish *Craspedacusta sowerbii* (Lankester, 1880) in Southern Hemisphere lakes (Chile) and the potential role of the zooplankton diet. Austral Ecol..

[40] Lüskow F., Boersma M., López-González P.J., Pakhomov E.A. (2022). Genetic variability, biomass parameters, elemental composition and energy content of the non-indigenous hydromedusa *Craspedacusta sowerbii* in North America. J. Plankton Res..

[41] Lüskow F., López-González P.J., Pakhomov E.A. (2021). Freshwater jellyfish in northern temperate lakes: *Craspedacusta sowerbii* in British Columbia, Canada. Aquat. Biol..

[42] Marchessaux G., Bejean M. (2020). Growth and ingestion rates of the freshwater jellyfish *Craspedacusta sowerbii*. J. Plankton Res..

[43] Sreeram M.P., Prasad R., Sreekumar K.M., Raju A.K., Augustina T.A.X., Lüskow F., Saravanan R. (2024). Post-flooding blooms of the non-indigenous freshwater jellyfish *Craspedacusta sowerbii* Lankester, 1880 in Kollam District of Kerala, India. J. Plankton Res..

[44] Lucas K., Colin S.P., Costello J.H., Katija K., Klos E. (2013). Fluid Interactions That Enable Stealth Predation by the Upstream-Foraging Hydromedusa *Craspedacusta sowerbyi*. Biol. Bull..

[45] Xu D., Xie Y., Li J. (2022). Toxic effects and molecular mechanisms of sulfamethoxazole on *Scenedesmus obliquus*. Ecotoxicol. Environ. Saf..

[46] Diogo B.S., Rodrigues S., Golovko O., Antunes S.C. (2024). From bacteria to fish: Ecotoxicological insights into sulfamethoxazole and trimethoprim. Environ. Sci. Pollut. Res..

[47] Felis E., Kalka J., Sochacki A., Kowalska K., Bajkacz S., Harnisz M., Korzeniewska E. (2020). Antimicrobial pharmaceuticals in the aquatic environment—Occurrence and environmental implications. Eur. J. Pharmacol..

[48] Genchi G., Sinicropi M.S., Lauria G., Carocci A., Catalano A. (2020). The Effects of Cadmium Toxicity. Int. J. Environ. Res. Public Health.

[49] Zhou J., Yun X., Wang J., Li Q., Wang Y., Zhang W., Fan Z. (2024). Biological toxicity of sulfamethoxazole in aquatic ecosystem on adult zebrafish (*Danio rerio*). Sci. Rep..

[50] Lin T., Yu S., Chen Y., Chen W. (2014). Integrated biomarker responses in zebrafish exposed to sulfonamides. Environ. Toxicol. Pharmacol..

[51] Roy D., Mitra A., Sen B.M., Homechaudhuri S. (2024). Biochemical Responses in Zebra Fish (*Danio rerio*) on Acute Cadmium Exposure Under Temperature Variations. Proc. Zool. Soc..

[52] Yuan W., Liang Y., Xia X., Xie Y., Lan S., Li X. (2018). Protection of *Danio rerio* from cadmium (Cd^2+^) toxicity using biological iron sulfide composites. Ecotoxicol. Environ. Saf..

[53] Tang K., Cao X., Geng X., Huang W., Liu H., Yan Z., Wu Z., Yang C., Tang J., Zhou Z. (2025). Microbiome dysbiosis and decreased survival in coral larvae exposed to environmentally relevant concentrations of nanoplastics and sulfamethoxazole. J. Hazard. Mater..

[54] Nykolay A., Shahid A. (2019). Immortal Hydra as a Model Organism for Metal Toxicity Studies. Sci. McMaster Undergrad. Sci. J..

[55] Murugadas A., Mahamuni D., Nirmaladevi S.D., Thamaraiselvi K., Thirumurugan R., Akbarsha M.A. (2019). Hydra as an alternative model organism for toxicity testing: Study using the endocrine disrupting chemical Bisphenol A. Biocatal. Agric. Biotechnol..

[56] Howe P.L., Reichelt-Brushett A.J., Clark M.W. (2014). Effects of Cd, Co, Cu, Ni and Zn on asexual reproduction and early development of the tropical sea anemone *Aiptasia pulchella*. Ecotoxicology.

[57] Wu X., Liao H., Zhang X., Ma Z., Fu Z. (2025). Unraveling the Impact of Microplastic–Tetracycline Composite Pollution on the Moon Jellyfish *Aurelia aurita*: Insights from Its Microbiome. Microorganisms.

[58] Xu W., Ahmed W., Mahmood M., Li W., Mehmood S. (2023). Physiological and biochemical responses of soft coral *Sarcophyton trocheliophorum* to doxycycline hydrochloride exposure. Sci. Rep..

[59] Elran R., Raam M., Kraus R., Brekhman V., Sher N., Plaschkes I., Chalifa-Caspi V., Lotan T. (2014). Early and late response of *Nematostella vectensis* transcriptome to heavy metals. Mol. Ecol..

[60] Morabito R., Dossena S., La Spada G., Marino A. (2014). Heavy metals affect nematocysts discharge response and biological activity of crude venom in the jellyfish *Pelagia noctiluca* (Cnidaria, Scyphozoa). Cell. Physiol. Biochem..

[61] Gao C.-H., Cao H., Ju F., Xiao K.-Q., Cai P., Wu Y., Huang Q. (2021). Emergent transcriptional adaption facilitates convergent succession within a synthetic community. ISME Commun..

[62] Wang H., Xu Y., Zhang Z., Zhang G., Tan C., Ye L. (2024). Development and application of transcriptomics technologies in plant science. Crop. Des..

[63] Jing L., Wang H., Xia S., Shao Q. (2025). Wnt/Ca(2+) signaling: Dichotomous roles in regulating tumor progress (Review). Oncol. Lett..

[64] Sanchez-Collado J., Lopez J.J., Jardin I., Salido G.M., Rosado J.A., Pedersen S.H.F. (2021). Cross-Talk Between the Adenylyl Cyclase/cAMP Pathway and Ca^2+^ Homeostasis. Reviews of Physiology, Biochemistry and Pharmacology.

[65] Schmidt M.F., Gan Z.Y., Komander D., Dewson G. (2021). Ubiquitin signalling in neurodegeneration: Mechanisms and therapeutic opportunities. Cell Death Differ..

[66] Dantuma N.P., Bott L.C. (2014). The ubiquitin-proteasome system in neurodegenerative diseases: Precipitating factor, yet part of the solution. Front. Mol. Neurosci..

[67] Ajoolabady A., Pratico D., Bahijri S., Tuomilehto J., Uversky V.N., Ren J. (2025). Hallmarks of cellular senescence: Biology, mechanisms, regulations. Exp. Mol. Med..

[68] Minchin D., Caffrey J.M., Haberlin D., Germaine D., Walsh C., Boelens R., Doyle T.K. (2016). First observations of the freshwater jellyfish *Craspedacusta sowerbii* Lankester, 1880 in Ireland coincides with unusually high water temperatures. Bioinvasions Rec..

[69] Schifani E., Viviano A., Viviano R., Naselli-Flores L., Marrone F. (2019). Different lineages of freshwater jellyfishes (Cnidaria, Olindiidae, *Craspedacusta*) invading Europe: Another piece of the puzzle from Sicily, Italy. Limnology.

[70] Seçer B. (2025). New locality records of invasive freshwater jellyfish *Craspedacusta sowerbii* (Lankester, 1880) in Türkiye. Limnol. Freshw. Biol..

[71] Moore J.P., Green H.C., Stewart D.J., Lüskow F., Wilder M.L. (2025). Invasive freshwater jellyfish (*Craspedacusta* cf. *sowerbii*) in the Hudson River basin, NYS: Comparisons of detection methods. Hydrobiologia.

