# Supplementary material for: Physiological and Transcriptomic Responses of the Freshwater Hydrozoan Craspedacusta sowerbii to Acute Antibiotic and Cadmium Exposure
Source: Biology (Basel). 2026 Jan 21;15(2):193. doi: 10.3390/biology15020193 (PMC12837586; doi:10.3390/biology15020193)
Supplement: Supplementary file 1 [file biology-15-00193-s001.zip › Supplementary File/File S1.pdf]

Biology

Manuscript ID: biology-4050720

Title: Physiological and Transcriptomic Responses of the Freshwater Hydrozoan *Craspedacusta sowerbii* to Antibiotic and Heavy Metal Pollution: Insights into Adaptive Mechanisms and Ecological Risks

Authors: Hailong Yan, Yu Wang, Yufan He, Jinglong Wang, Mengyao Wu, Shang Shi, Jianing Shi, Jingjing Guo, Nicola Fohrer, Jianguang Qin, and Yuying Li

Supplementary Figure S1-S3

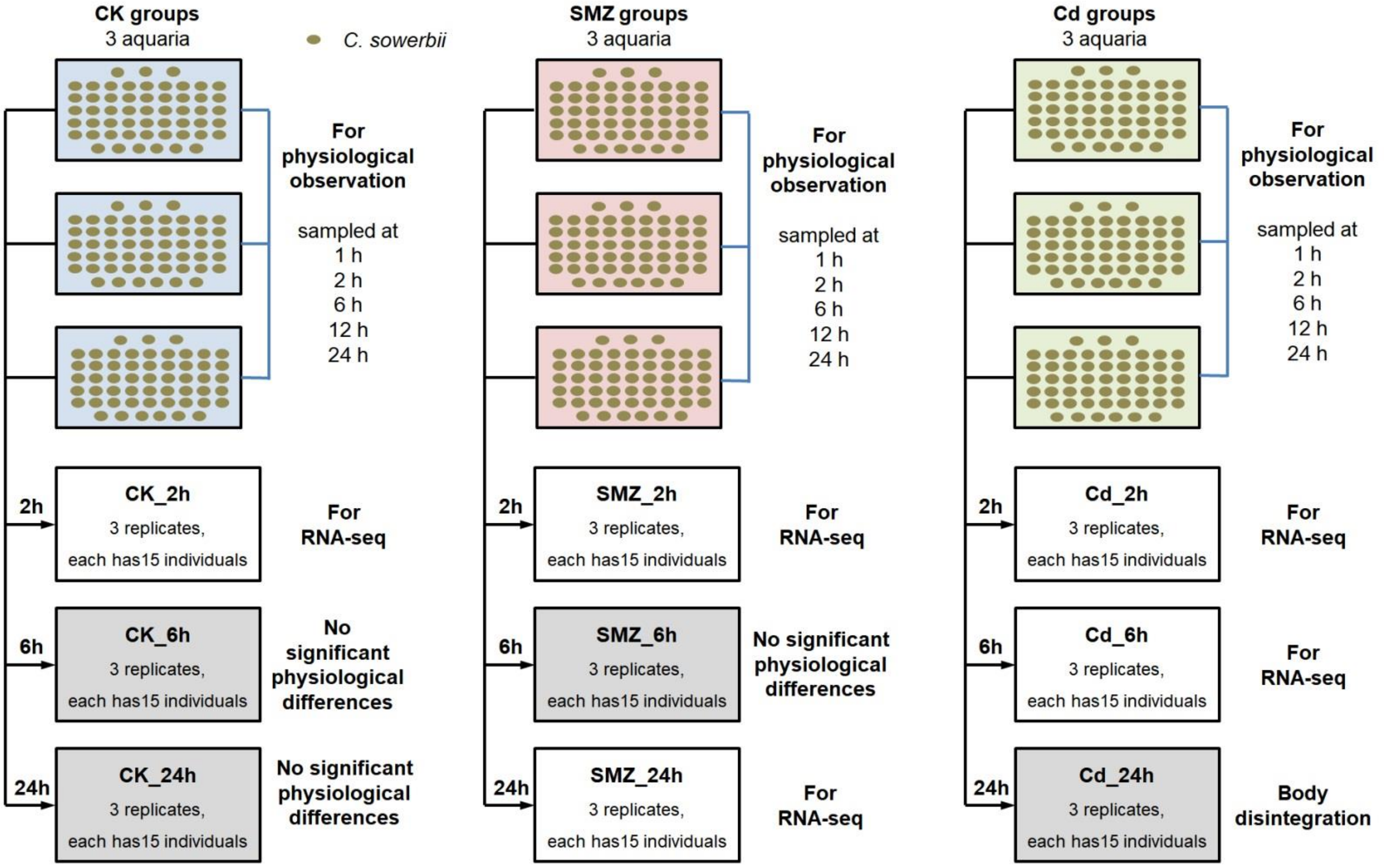

Figure S1. Schematic diagram illustrating the experimental sampling strategy. The experiment comprised nine groups: three CK groups, three SMZ-treated groups, and three Cd-treated groups, each group initially contained approximately 50 individuals of *C. sowerbii*. Following the addition of SMZ or Cd, 15 individuals were randomly sampled from each group at 2, 6, and 24 hours post-treatment for subsequent RNA-seq analysis. This sampling strategy generated three biological replicates for each of the nine treatment-time combinations: CK_2h, CK_6h, CK_24h, SMZ_2h, SMZ_6h, SMZ_24h, Cd_2h, Cd_6h, and Cd_24h. Among the collected samples, CK_6h and CK_24h showed no significant physiological differences with CK_2h, SMZ_6h showed no significant physiological differences with SMZ_2h, and the individuals of *C. sowerbii* in Cd_24h showed severe body disintegration. Therefore, a subset comprising CK_2h (naming as CK in RNA-seq analysis), SMZ_2h, SMZ_24h, Cd_2h, and Cd_6h was selected for RNA-seq analysis to assess transcriptional responses to SMZ and Cd exposures.

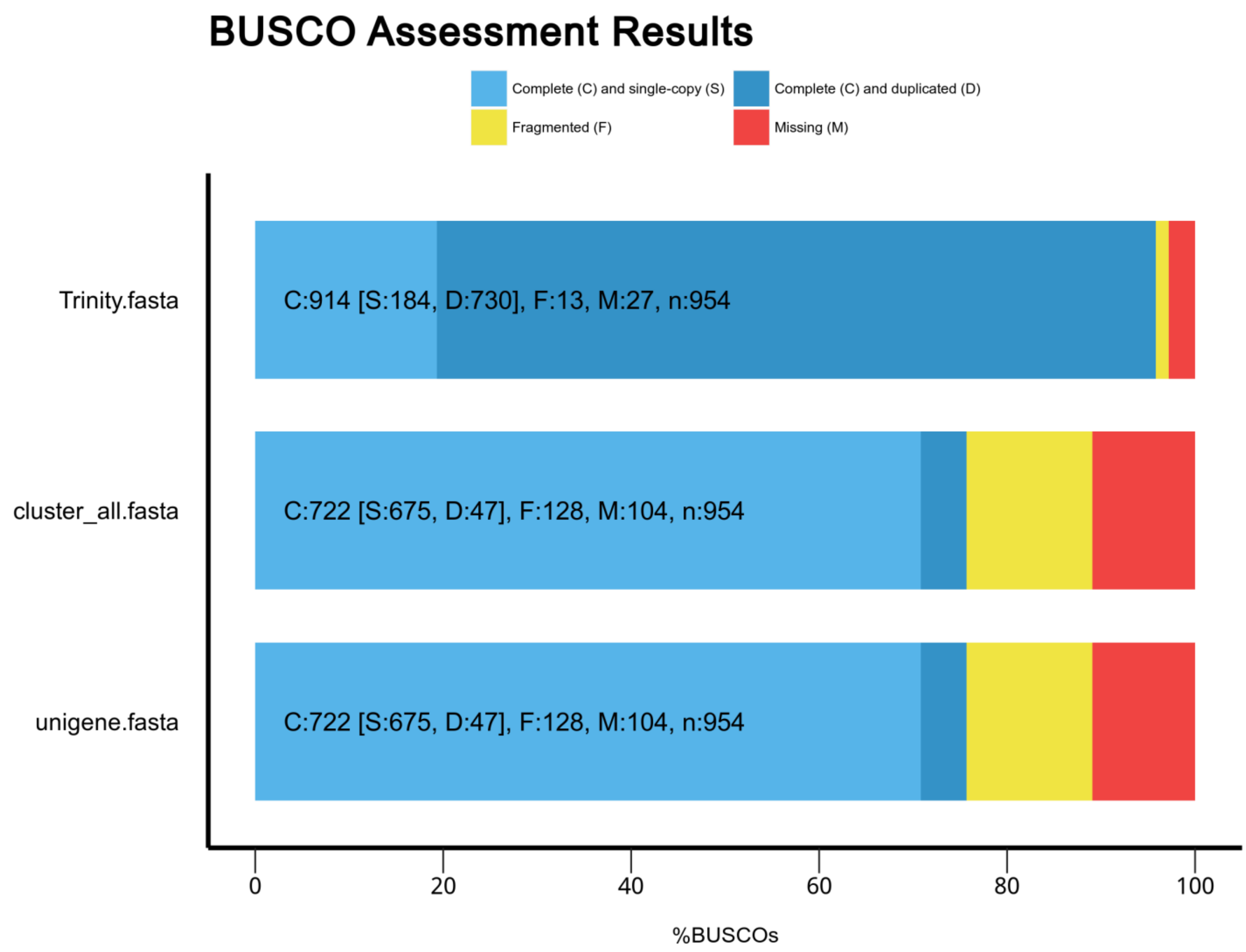

Figure S2. The BUSCO assessment result. BUSCO (Benchmarking Universal Single-Copy Orthologs) assessment evaluates transcriptome assemblies using a curated database of single-copy orthologous genes, in combination with tools such as *tblastn*, *Augustus*, and *HMMER*, to assess the completeness of assembled transcripts. In this study, BUSCO was applied to evaluate the assembly quality of *Trinity.fasta*, *unigene.fa*, and *cluster.fasta*. The accuracy and completeness of the assemblies were assessed based on the proportion of matched BUSCO genes and their completeness scores.

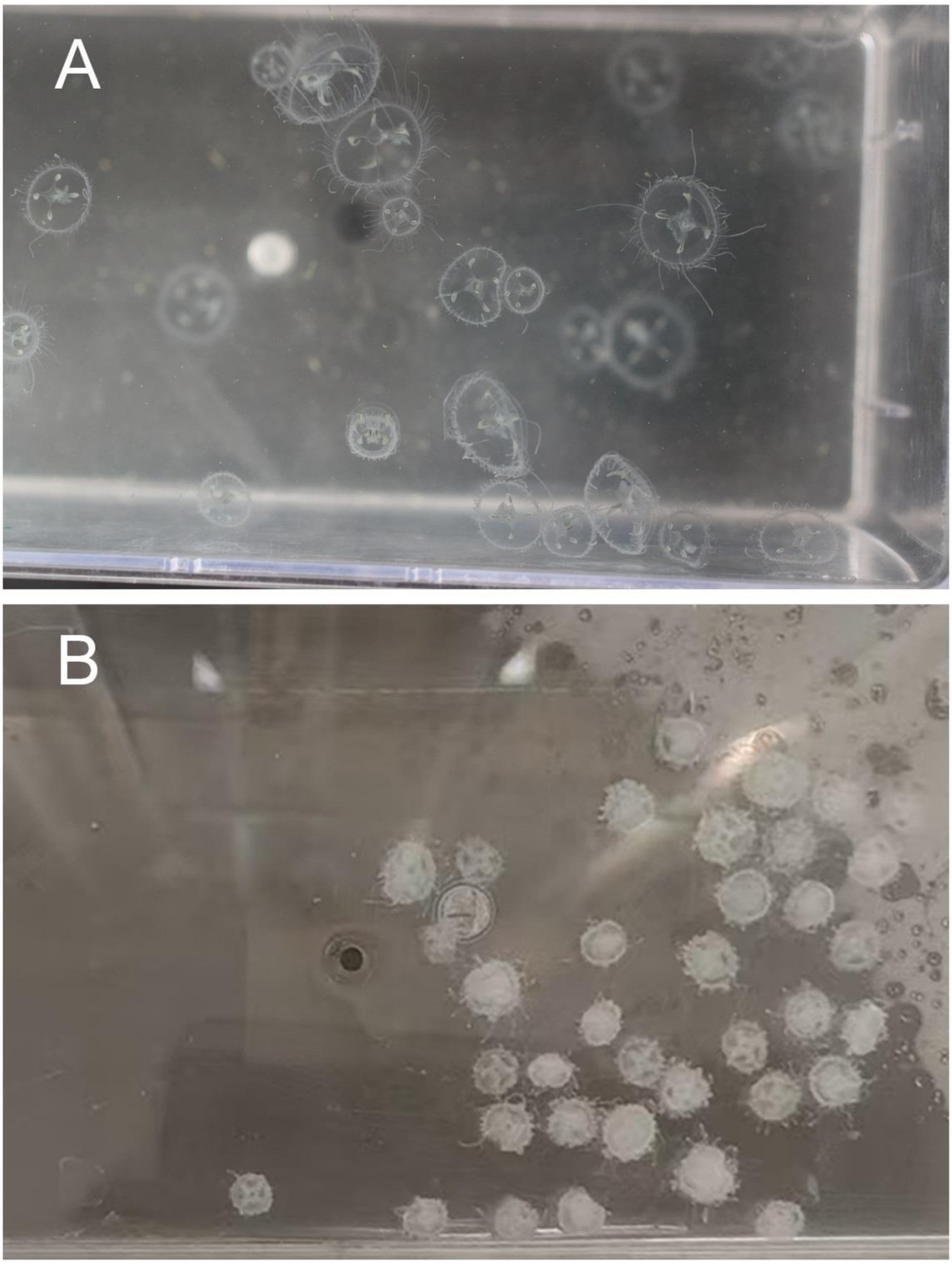

Figure S3. Following 24 hours of Cd treatment, the bodies of *C. sowerbii* ceased movement and disintegrated into fragments. Control group of *C. sowerbii*, which was cultured using pond water (A). Cd-treated group of *C. sowerbii*, which was cultured using pond water supplemented with 10 μM cadmium sulfate, sampled 24 hours post-treatment.
