# Supplementary material for: Physiological and Transcriptomic Responses of the Freshwater Hydrozoan Craspedacusta sowerbii to Acute Antibiotic and Cadmium Exposure
Source: Biology (Basel). 2026 Jan 21;15(2):193. doi: 10.3390/biology15020193 (PMC12837586; doi:10.3390/biology15020193)
Supplement: Supplementary file 1 [file biology-15-00193-s001.zip › Supplementary File/File S3.pdf]

Biology

Manuscript ID: biology-4050720

Title: Physiological and Transcriptomic Responses of the Freshwater Hydrozoan *Craspedacusta sowerbii* to Antibiotic and Heavy Metal Pollution: Insights into Adaptive Mechanisms and Ecological Risks

Authors: Hailong Yan, Yu Wang, Yufan He, Jinglong Wang, Mengyao Wu, Shang Shi, Jianing Shi, Jingjing Guo, Nicola Fohrer, Jianguang Qin, and Yuying Li

Supplementary File S3. GO and KEGG functional enrichment.

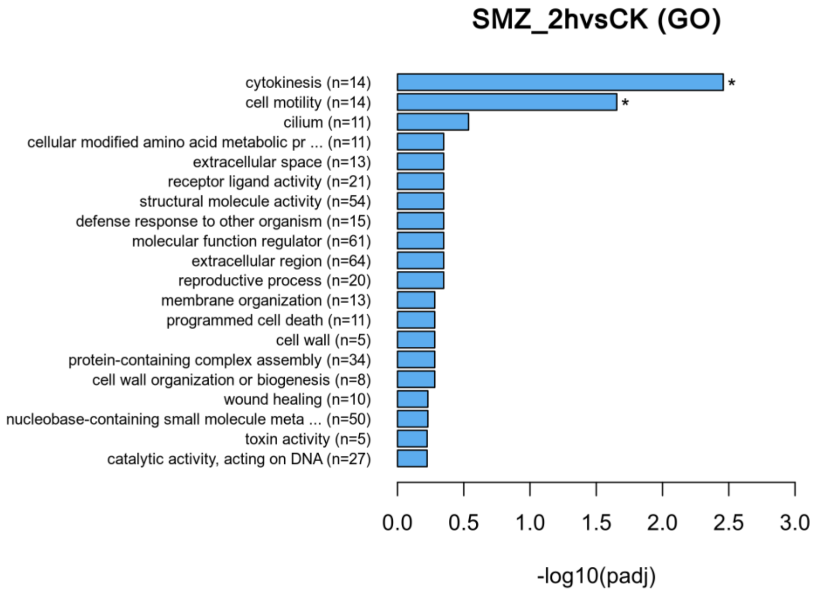

GO enrichment of SMZ_2h vs CK (All)

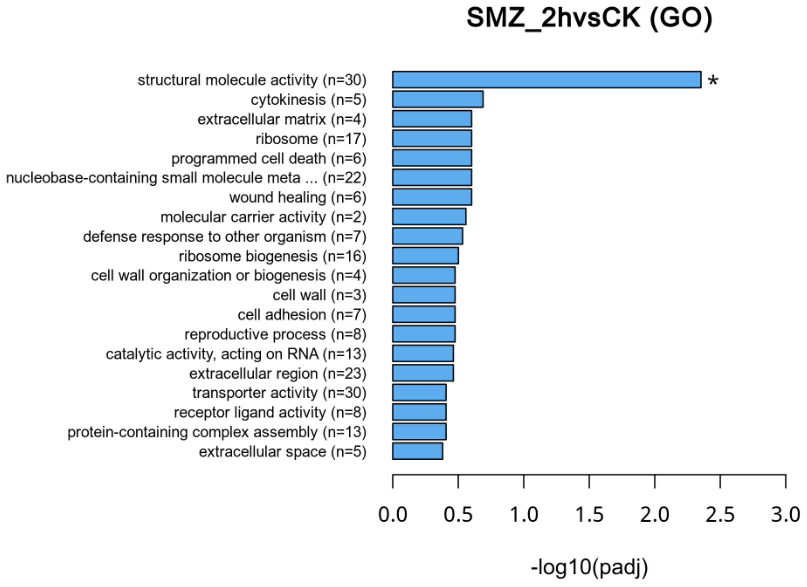

GO enrichment of SMZ_2h vs CK (Up)

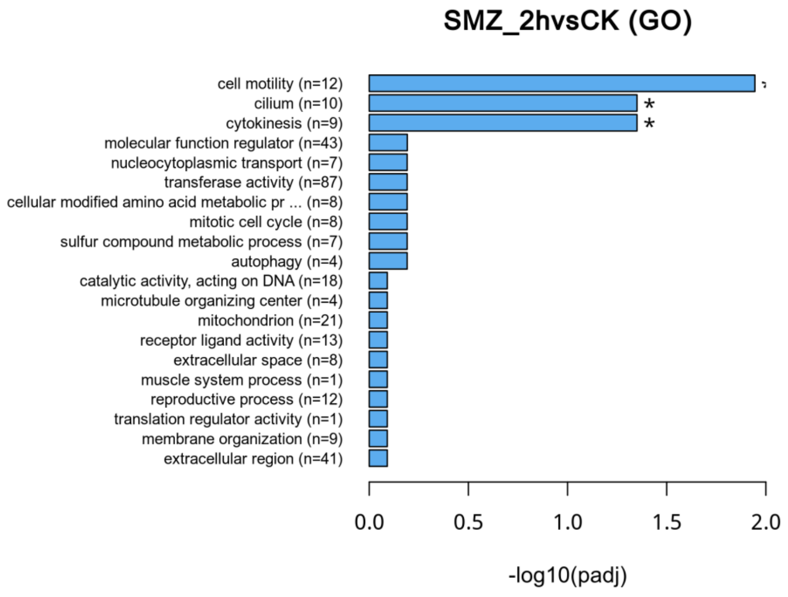

GO enrichment of SMZ_2h vs CK (Down)

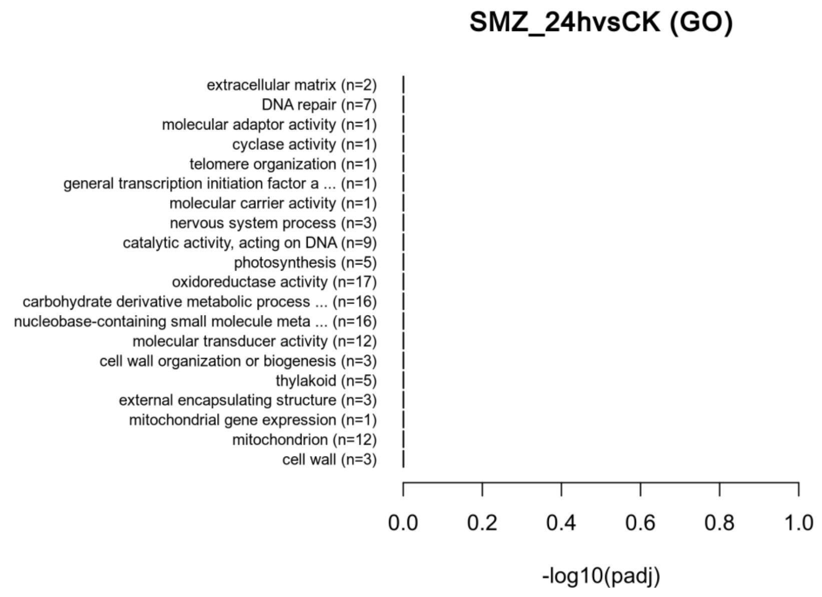

GO enrichment of SMZ_24h vs CK (All)

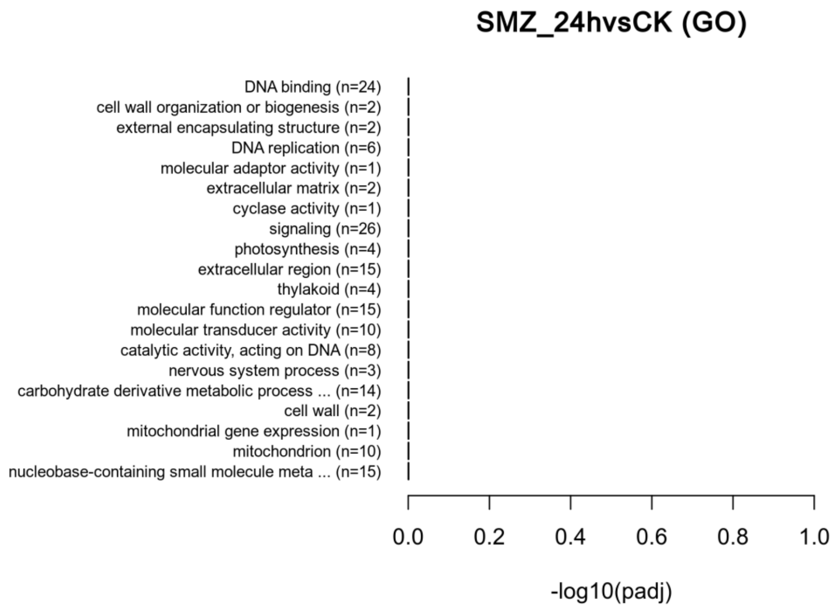

GO enrichment of SMZ_24h vs CK (Up)

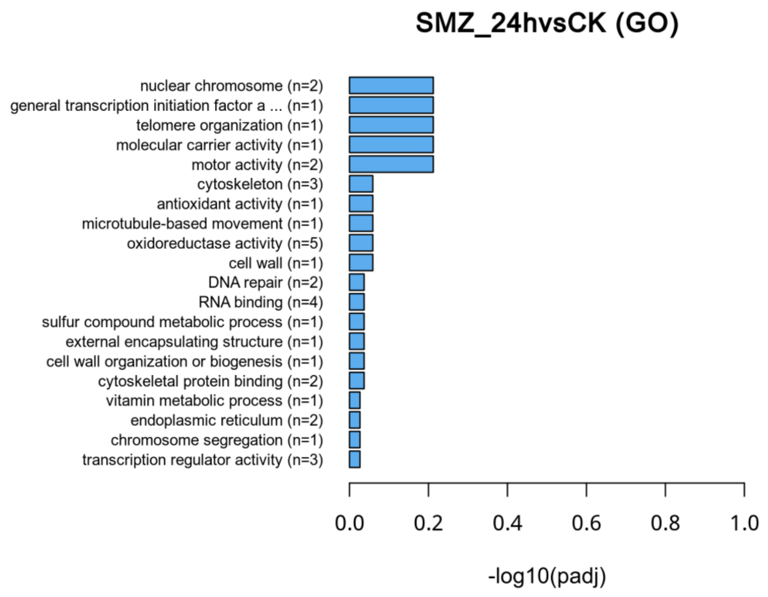

GO enrichment of SMZ_24h vs CK (Down)

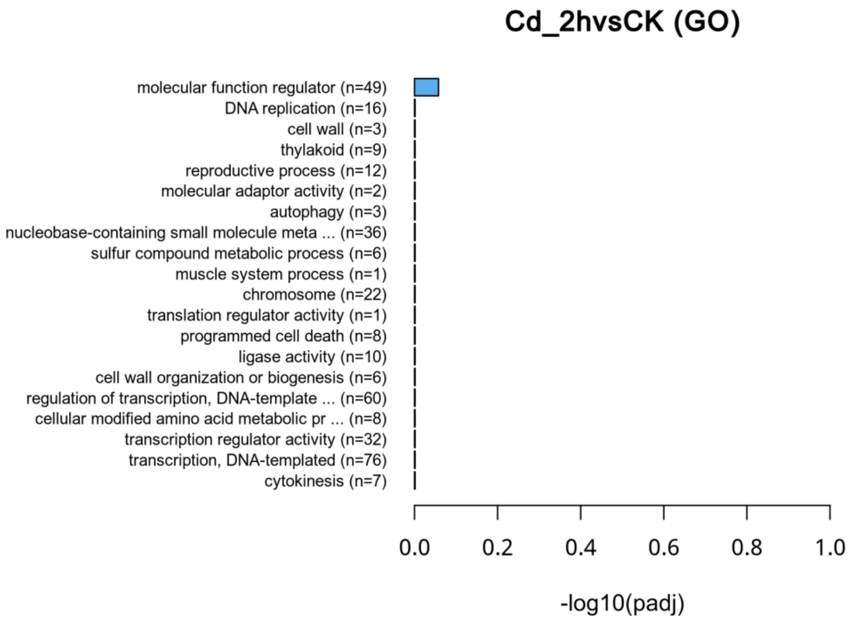

GO enrichment of Cd_2h vs CK (All)

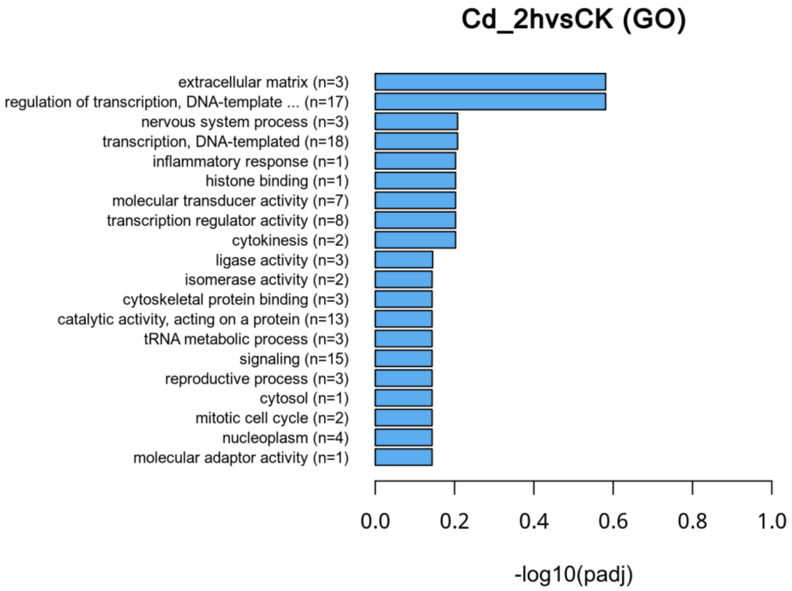

GO enrichment of Cd_2h vs CK (Up)

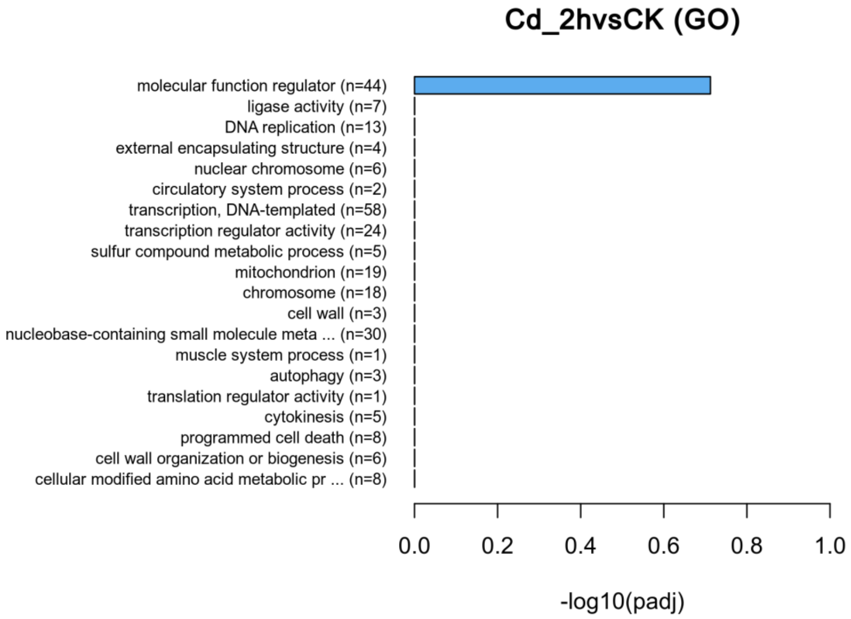

GO enrichment of Cd_2h vs CK (Down)

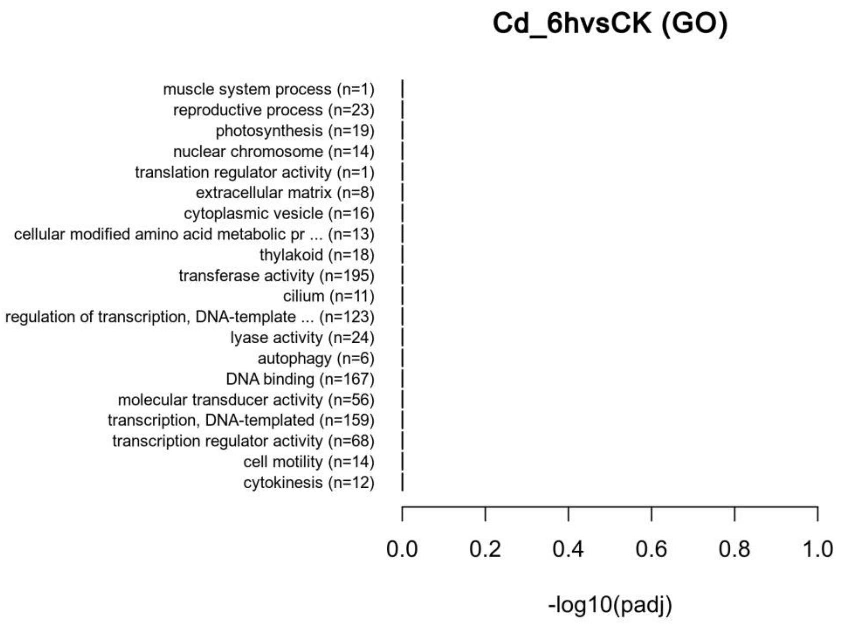

GO enrichment of Cd_6h vs CK (All)

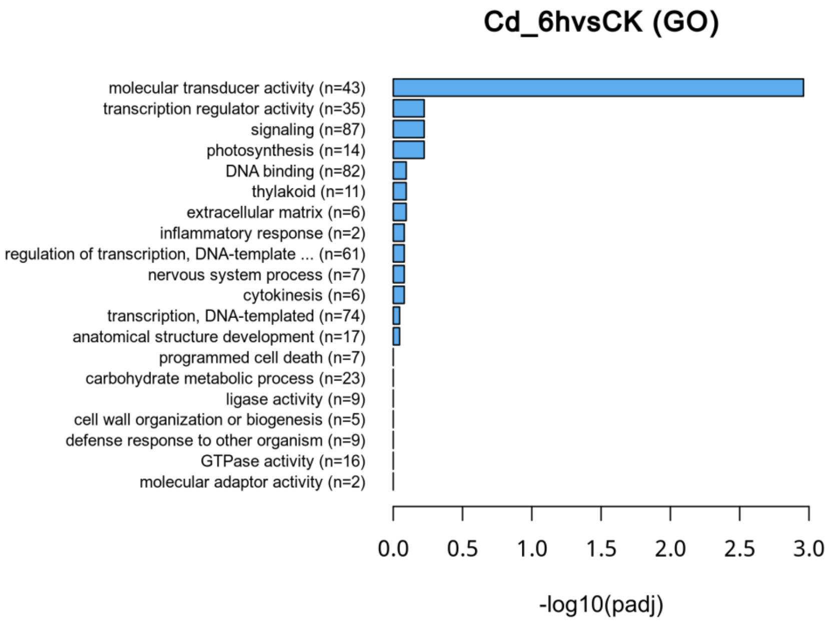

GO enrichment of Cd_6h vs CK (Up)

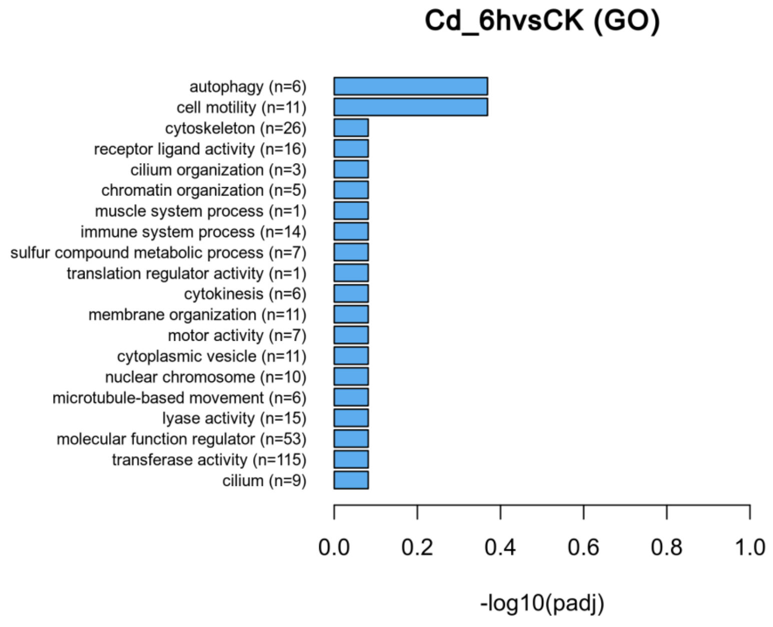

GO enrichment of Cd_6h vs CK (Down)

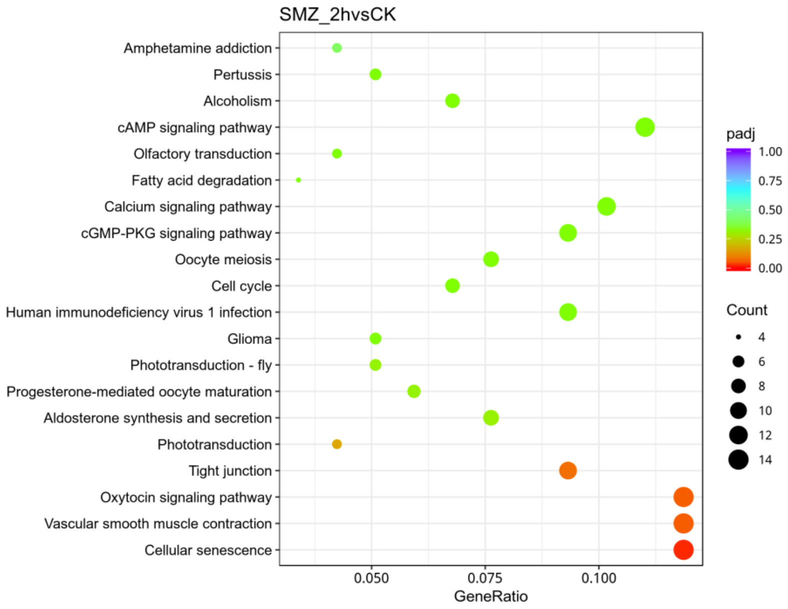

KEGG enrichment of SMZ_2h vs CK (All)

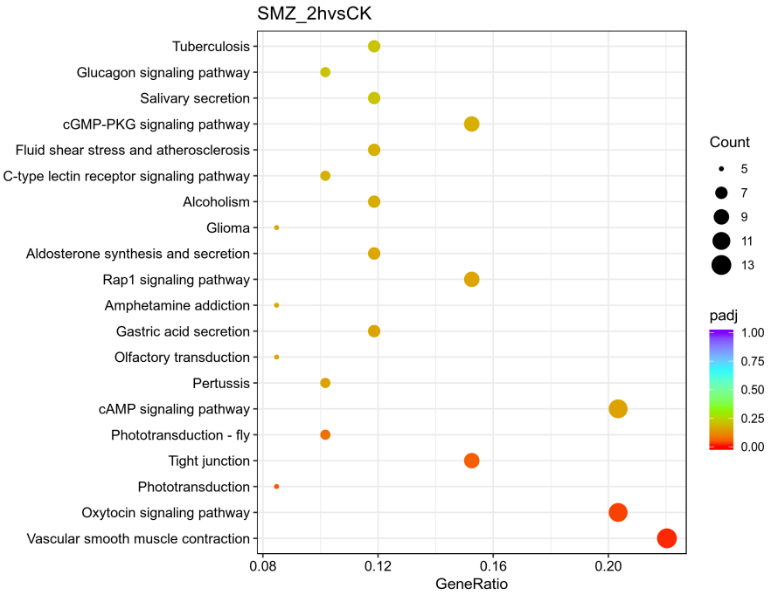

KEGG enrichment of SMZ_2h vs CK (Up)

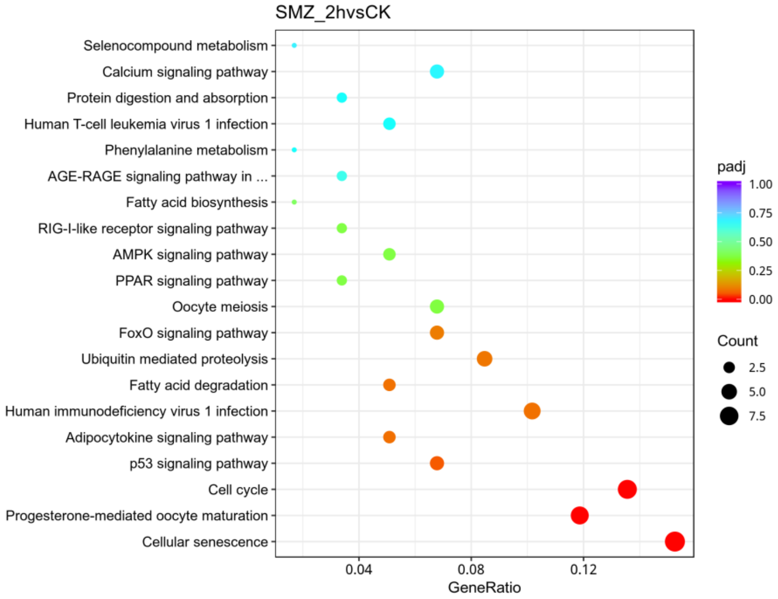

KEGG enrichment of SMZ_2h vs CK (Down)

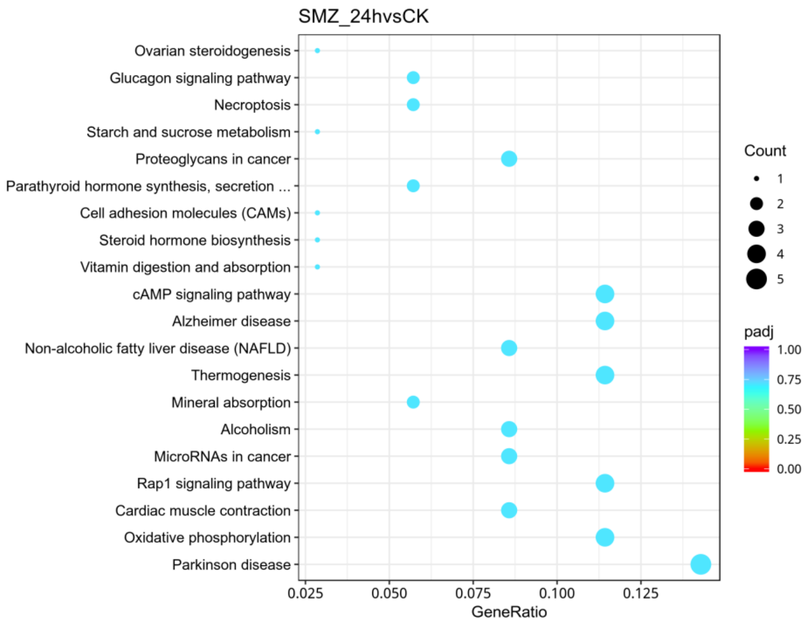

KEGG enrichment of SMZ_24h vs CK (All)

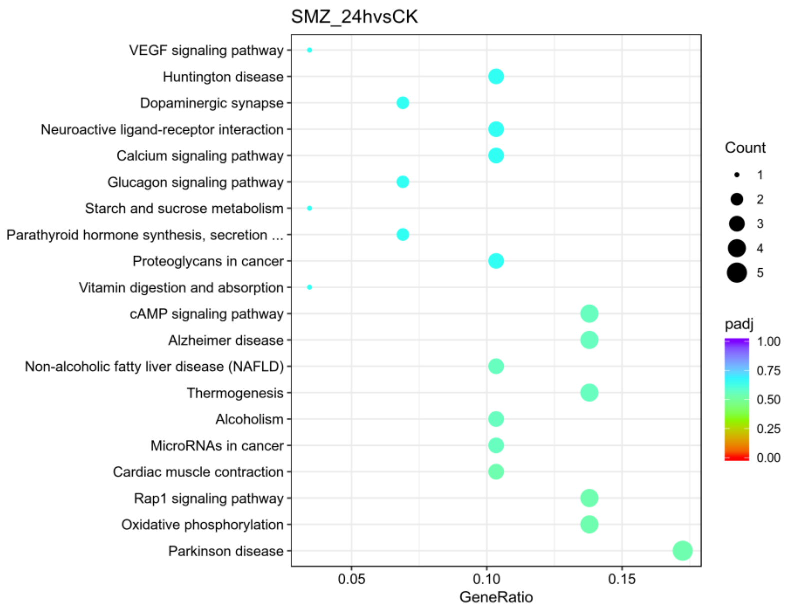

KEGG enrichment of SMZ_24h vs CK (Up)

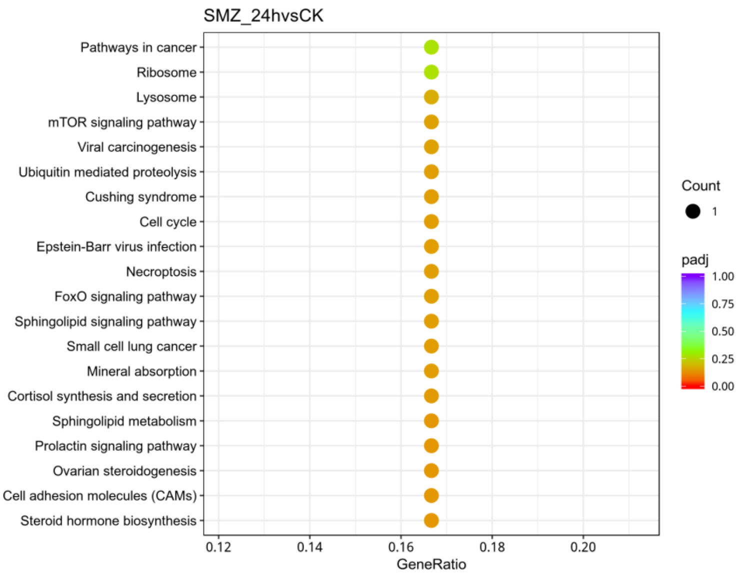

KEGG enrichment of SMZ_24h vs CK (Down)

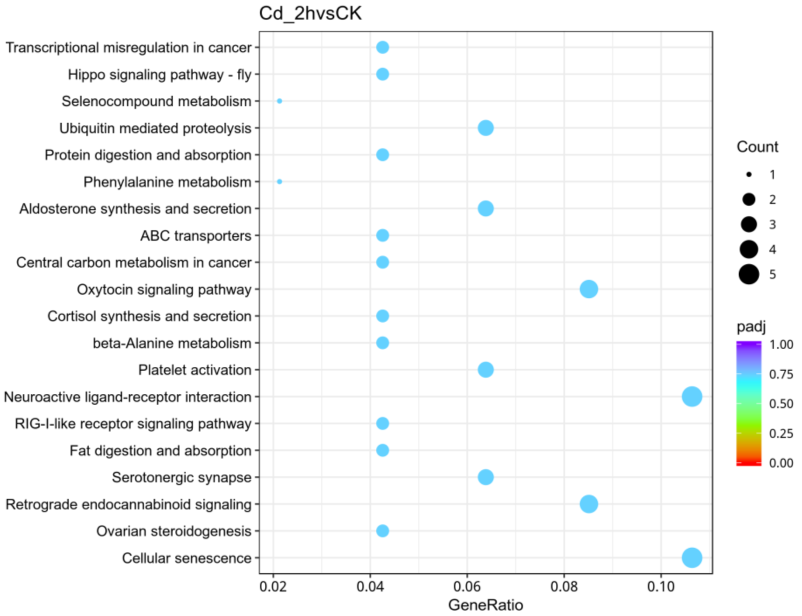

KEGG enrichment of Cd_2h vs CK (All)

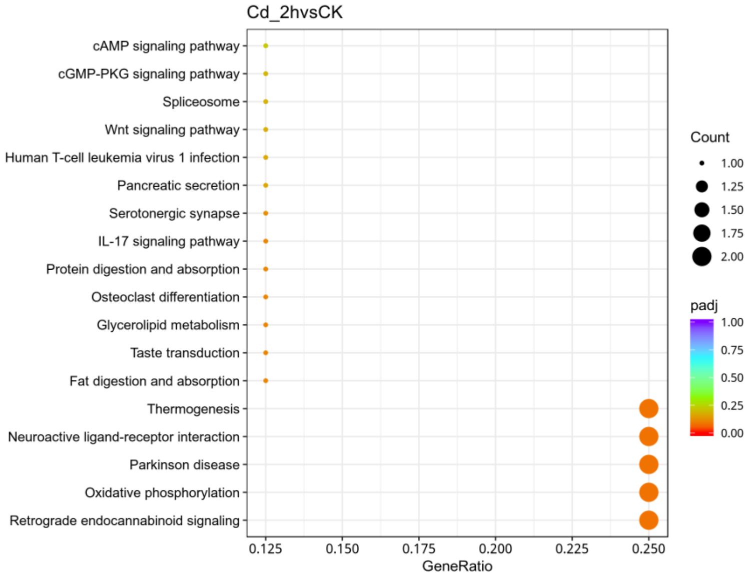

KEGG enrichment of Cd_2h vs CK (Up)

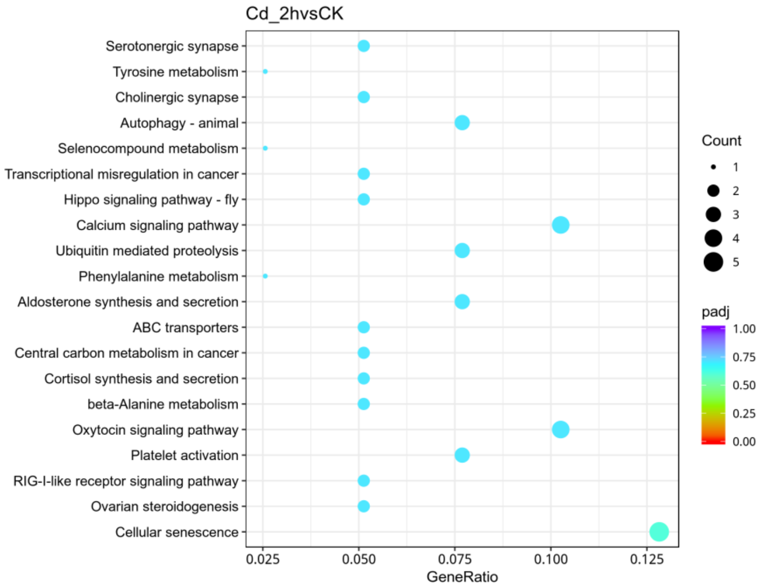

KEGG enrichment of Cd_2h vs CK (Down)

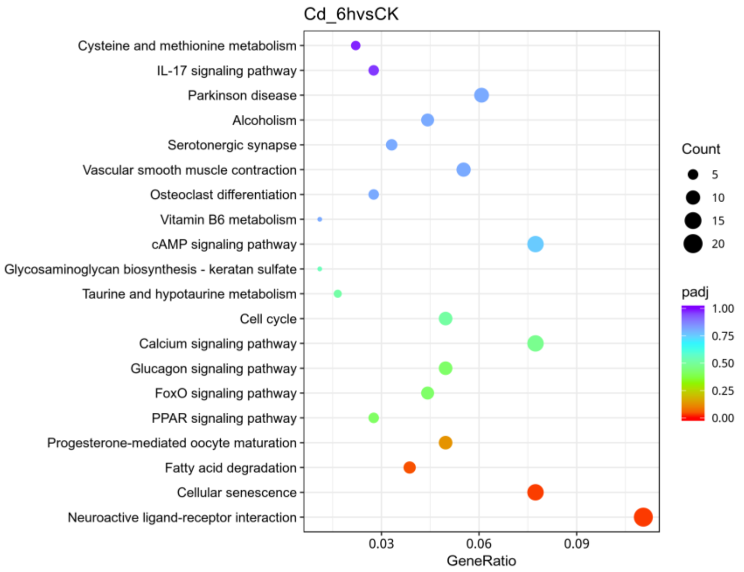

KEGG enrichment of Cd_6h vs CK (All)

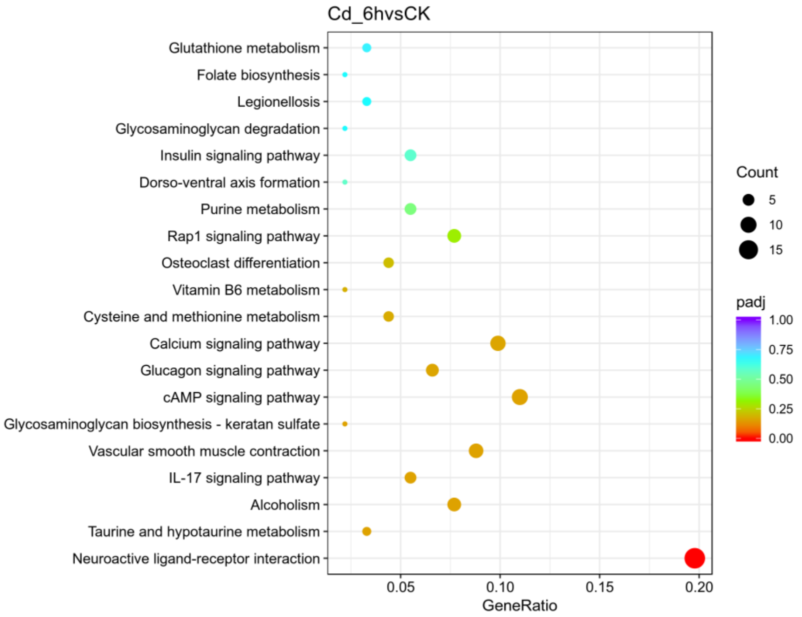

KEGG enrichment of Cd_6h vs CK (Up)

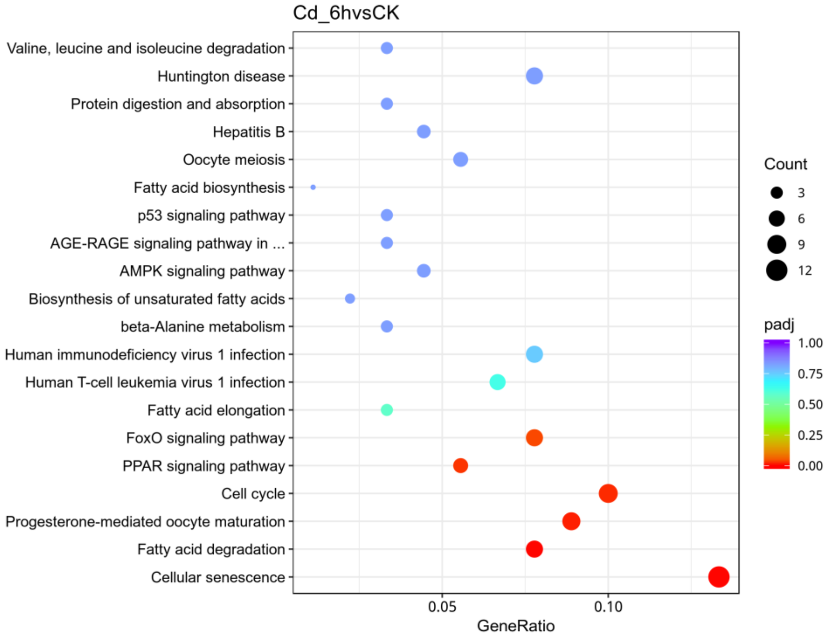

KEGG enrichment of Cd_6h vs CK (Down)
